# Dissecting the dissection laboratory: Elements for designing a human anatomy laboratory: The Human Anatomy and Physiology Society (HAPS)—Anatomical Donor Stewardship (ADS) Committee, Lab Resources Subcommittee

**DOI:** 10.1002/ase.70344

**Published:** 2026-09-16

**Authors:** Rhiannon E. Robinson, Abbey Breckling, Danielle N. Edwards, Nicole Geske, Burhan Gharaibeh, Bobbie J. Leeper, Molly Ostwald, Mary Schilling, Bridgett Severt, Kelsey Stevens, J. P. Swigart, Jonathan J. Wisco, Melissa Zolnierz, Jeremy J. Grachan

**Affiliations:** ^1^ Department of Anatomy & Neurobiology Boston University Chobanian & Avedisian School of Medicine Boston Massachusetts USA; ^2^ Department of Anatomy and Cell Biology, College of Medicine University of Illinois at Chicago Chicago Illinois USA; ^3^ Department of Anatomical Sciences and Neurobiology University of Louisville School of Medicine Louisville Kentucky USA; ^4^ Department of Radiology, College of Osteopathic Medicine Michigan State University East Lansing Michigan USA; ^5^ Department of Biological Sciences University of Pittsburgh Pittsburgh Pennsylvania USA; ^6^ Biology and Physician Assistant Programs Seton Hill University Greensburg Pennsylvania USA; ^7^ Department of Natural Sciences Arapahoe Community College Littleton Colorado USA; ^8^ Department of Biological Sciences Northern Kentucky University Highland Heights Kentucky USA; ^9^ Department of Neuroscience, Cell Biology, and Physiology Wright State University & Boonshoft School of Medicine Dayton Ohio USA; ^10^ Department of Exercise and Health Sciences Briar Cliff University Sioux City Iowa USA; ^11^ Department of Biomedical and Translational Sciences Carle Illinois College of Medicine Urbana Illinois USA; ^12^ Department of Pathology and Anatomical Sciences Kansas City University Joplin Missouri USA; ^13^ Department of Medicine Rutgers New Jersey Medical School Newark New Jersey USA

**Keywords:** anatomy laboratory design, anatomy laboratory resources, body donors, cadavers, dissection

## Abstract

The human anatomy dissection laboratory is a critical component of anatomical and medical education. Development, maintenance, and stocking of these laboratory spaces have unique demands, with all of the challenges in running a laboratory with Biosafety Laboratory Level 2 (BSL‐2) classification, housing a large volume of tissues saturated with volatile chemicals, and safeguarding human beings all within the same space. At times, educators or laboratory managers may be tasked with helping to design a new anatomy laboratory from scratch, to give input on the renovation of an existing laboratory space (which may or may not have been used with human anatomy donors previously), or may inherit a human anatomy laboratory space as their own to manage. This Guide was written by members of the Human Anatomy and Physiology Society (HAPS) Anatomical Donor Stewardship (ADS) Lab Resources Subcommittee to guide those developing new anatomy dissection laboratories or working in existing ones.

## INTRODUCTION

The human anatomy dissection laboratory is often considered to be an essential component of anatomical science education.^1^, ^2^ Here, students are able to gain important insight into the 3‐dimensional spatial relationships of structures within the body, are able to integrate didactic learning and cross‐sectional anatomy with true anatomy, and appreciate the extent and breadth of normal anatomical variation.^3^, ^4^ Furthermore, human anatomy dissection provides instructors with an unmatched opportunity to incorporate humanistic elements, fostering professional development toward empathetic, mindful future anatomists, and clinicians.^5^, ^6^

Designing or reinvigorating an anatomy dissection laboratory requires careful consideration of spatial constraints, workflow, safety, and regulatory compliance. Unique to the anatomy laboratory, the need to house embalmed humans for long periods of time creates a burden of vigilance on personnel that other laboratories do not require. The demands of running this type of laboratory are unique; therefore, standard guidance for operating other types of research or educational laboratories does not fully apply.^7^ Yet despite the central role these laboratories play in anatomy education and the unusual demands they place upon staff and faculty, there is insufficient literature detailing comprehensive, practical guidance in the literature regarding designing and implementing a human anatomy laboratory that could be found at the time of writing. There are a few publications describing considerations in designing or redesigning human dissection laboratories for specific institutional needs^7^, ^8^, ^9^, ^10^ and some publications focus on specific aspects of the anatomy laboratory design process, such as the financial considerations,^11^ the ventilation system,^12^, ^13^ or the technology needs.^14^, ^15^ The Human Anatomy and Physiology Society (HAPS) and the American Association for Anatomy (AAA) also both provide recommended considerations as well as example laboratory designs on their respective websites for their members.^16^, ^17^

The HAPS Anatomical Donor Stewardship Committee (ADS) Lab Resources Subcommittee aims to compile the practical knowledge of its subject‐matter experts, accumulated over years of experience. This Guide addresses elements of laboratory design, including (but not limited to) evaluating available space, infrastructural considerations, stocking and replenishment, and sustainability. It provides a practical framework adaptable to a wide range of institutional environments and intends to help educators develop or maintain functional, compliant, and thoughtfully planned dissection laboratories. While this Guide is largely logistical in nature, it also offers some best‐practice suggestions for student management, workflow, ergonomics, and infrastructure management.

### Basics of laboratory design

#### Space and organization

Anatomy laboratories vary tremendously between institutions. They may be brand‐new, purpose‐built, or retrofitted from a space that may or may not have been designed as laboratory space. The size of the laboratory is an obvious constraint regarding how many anatomy donors and how many students can be accommodated in a laboratory. A given area will likely have a limit to the number of living occupants in that space set by the fire department, though realistically the desired maximum capacity for an anatomy laboratory will be less than that. A human anatomy donor lays prone on a table larger than themselves, surrounded by students who need elbow room to dissect, who are walking around with sharps, and all while maintaining sufficient air turnover and temperature control that the room remains comfortable. When considering how many dissection tables can fit into a room, there should be ~4 feet (~1.25 m) between tables surrounded by dissectors to allow people to move around the room freely without bumping into others. Consider that this may require creative organization or arrangement if there are load‐bearing pillars in the room, visual obstructions between where tables must be and where the instructor plans to be, and more space may be required than this if your institution's accessibility department requires it.

Laptops, iPads, and other digital resources can play a useful supplementary role in donor‐based anatomy laboratories.^15^, ^18^, ^19^, ^20^ Resources at each donor table, if available, allow students to obtain anatomical reference information during laboratory sessions. For example, students can pull up the dissector to guide them through dissection and refer to pictures to aid in finding structures. Further, they can compare views seen in 3D digital models with real structures to help in identification on a specimen. For safe placement of these items, we suggest that each station be equipped with a small stainless steel table that is connected to an electrical source, to ethernet, or to other required ports. Signs that guide the students to not use while gloved can prevent the spread of embalming chemicals and biological material onto shared equipment.

Finally, while the physical layout and organization of an anatomy laboratory are foundational considerations, effective operation also depends on clear oversight and defined responsibility for safety, compliance, and daily function. Institutions may designate these responsibilities differently, and no single organizational model fits all programs. Oversight might be assigned to individuals with titles such as laboratory director or manager or to a faculty member who assumes an additional administrative role. These responsibilities are typically accompanied by institution‐specific training, most often coordinated through environmental health and safety (EHS) or comparable offices, to ensure that laboratory use remains aligned with regulatory requirements, best practices, and institutional policies.

#### Traffic flow

Next, consider the traffic flow of the room. Dissectors will arrive to the laboratory with clean clothes (wearing clean scrubs or bringing clean scrubs to change into) and likely will bring personal belongings that they will need to store (e.g., backpack and jacket). They will need to don personal protective equipment (PPE), enter the dissection area to work, clean up, remove PPE, wash up, and then leave the room. Ideally, an unobstructed path that naturally leads dissectors through these regions without having to cross over will be created by the organization of the room. Spaces where tissues reside are considered “dirty” areas and spaces without tissue are considered “clean”; these areas should be clearly labeled and delineated with tape on the ground and signage. Given this workflow, the area where dissectors immediately enter the laboratory, may be stashing their belongings, and where they will be donning PPE (if done inside of the laboratory) should be a devoted “clean” area. An in‐laboratory PPE donning area should be immediately adjacent to the “dirty” work area of the laboratory, and wearing of PPE in “clean” areas, once donned, should be prohibited. Dedicated washing areas for instruments, and storage of cleaning supplies for spills, wiping down tables, and refilling wetting solutions should all be housed inside the “dirty” area so dissectors do not have to leave and remove PPE every time they need one of these items. Before they exit, there should be a handwashing station and biohazard bins for students to remove PPE and wash their hands so as to re‐enter the “clean” area in as close to a state as they entered as possible.

#### General laboratory items

Human anatomy laboratories may use fresh donors, embalmed donors, or a combination of both. Laboratories that use embalmed donors are classified as Biosafety Level (BSL) 1,^21^ though individual institutions may prefer to treat these spaces as BSL‐2 and err on the side of extra safety. Laboratories that use fresh donors are treated as BSL‐2, which comes along with its own set of requirements, including (but not necessarily limited to):

- **Restricted Access:** A list of people with access should be kept at all times and revisited regularly to add and remove people who no longer need that access. Ideally, this access will be restricted with additional security measures such as requiring scanning of a badge or obtaining a key. Only those people granted access may be permitted into the laboratory. As permissions may change frequently, a digital list would be easiest to maintain.
- **Specialized Training:** To gain access to the laboratory, in addition to demonstrating a need to be there, all people entering must have passed BSL‐2 training.
- **PPE Requirements:** PPE must be worn at all times in the laboratory, including eye protection, face protection, and gloves. Open‐toed shoes and closed‐toe shoes with large openings are prohibited.
- **No Food/Drink:** No food or drink may be consumed in the laboratory. Relatedly, anything touching one's mucus membranes may also not be used (e.g., eye drops, gum, lip balm).
- **Biohazard‐Dedicated Waste Stream:** All waste that comes into contact with human tissue, or is used within the “dirty” space, must be disposed of in biohazard waste bins, lined with biohazard waste bags, all of which are clearly labeled as such and are destined for a dedicated waste stream. Details surrounding what “counts” for the biohazard waste stream will vary between institutions, and your institution's EHS department should advise this delineation.
- **Spill Kits:** Kits for cleaning up spills of hazardous chemicals or with biological materials (e.g., human fluids) must be cleaned up with specific protocols.
- **Handwashing Sinks:** Handwashing sinks must be provided at convenient locations in an area in or adjacent to a “dirty” area.
- **Eyewash Stations:** Eyewash stations must be provided and maintained to institutional standards to flush out the eyes in the event the eyes come into contact with any human fluids or laboratory chemicals.
- **Negative Airflow:** The air pressure in the laboratory should be lower than that of the surrounding spaces, so that air flows inwards into the laboratory whenever doors are opened, rather than outward into the public areas, for vapor exposure and biohazard risk control. This can be accomplished with adequate ventilation. *This is strongly recommended regardless of BSL level to avoid irritating fumes and odors from escaping into areas of general access (e.g., hallways)*.
- **Self‐Closing Doors:** Doors should close on their own to help maintain this negative pressure and to preserve restricted access.

Depending on what else the laboratory space is being used for aside from human dissection, the laboratory may also need to be equipped with biosafety cabinets and an autoclave.

#### Considerations for use of the guide

When designing and purchasing for a laboratory, it is important to distinguish between items that are essential versus non‐essential. As this is a team effort, consider the following:

- Is your team building or organizing a new laboratory from the ground up? *The physical space directs what can be done with it—designing it with flexibility, sustainability, and growth in mind affords the most opportunity for maximizing time and money spent and minimizing recurring costs*.
- Is your team working with a laboratory space with a new trajectory (space that is repurposed for human anatomy laboratory use)? *Identifying infrastructural limitations and requirements—such as air turnover requirements, drainage, and number and location of sinks—and understanding what can and cannot be changed early on will determine what “makeshift” infrastructural solutions will be needed (*e.g., *a standalone air purifier instead of one built into the HVAC system), which in turn will determine how you organize and stock the laboratory space*.
- Is your team taking responsibility for an existing human anatomy laboratory and tasked with purging materials, assessing the quality of materials, replacing materials, taking an inventory of supplies, revamping the laboratory space, etc.? *What strengths and weaknesses exist for the laboratory space as it stands, without changing it? Can your team identify a reason for why those weaknesses exist—and are they realistically changeable? What changes can be put in place that will be easily adapted to by other staff, faculty, and students who are accustomed to using the laboratory with minimal disruption to their work?*

In any case, many individuals may be responsible for materials management, purchasing, and directing renovations, and these groups of people may or may not overlap. In each of these roles, each person may have a different degree of autonomy, time, institutional support for their goals, funding, and regulatory constraints to work within. Other readers will be largely left to their own devices to execute the development of a laboratory, procurement of materials, and/or maintenance of an anatomy laboratory with little interference (but perhaps also little active support).

Major overarching considerations when choosing anything—from infrastructural items to small, single‐use disposable items—are as follows:

- What are the institutional, state, or federal requirements surrounding its use (e.g., Environmental Health and Safety department protocols, OSHA regulations, Fire Code capacity restrictions)?
- How does this affect the safety of all people in the room (e.g., sharps management, fume mitigation, ergonomic considerations)?
- Does this compromise donor dignity, ethical standards, or privacy? If the answer is “yes”, or “maybe” to any of the above—discard and reconsider your approach carefully. The HAPS ADS Ethics subcommittee works closely with the Lab Resources subcommittee to provide solutions for laboratory resources problems and can help rethink your strategy.

### Purpose of the guide

This Guide serves to assist instructors and laboratory personnel who are charged with building, maintaining, or selecting supplies for use in preserved whole‐body human anatomy donor dissection laboratories. While individual tissues (e.g., organs in buckets) and dry tissues (e.g., articulated skeletons, boxes of disarticulated bones) are not directly addressed in this Guide, some advice in this guide can still be useful for those components (e.g., embalming considerations, laboratory practical components). Advice regarding models or plasticized remains is excluded as outside of the scope of this Guide. The advice and information in this Guide come from the collective expertise of the HAPS ADS Lab Resources subcommittee, drawn from their experiences with laboratory maintenance and teaching with human donors.

The contents of this Guide are not intended to be exhaustive, nor should they be treated like an all‐encompassing checklist to be functional, nor is this list meant to be prescriptive or comprehensive. Each laboratory has unique needs, opportunities, and challenges; so, it is strongly recommended that the suggestions found in this Guide should be interpreted within the context of guidelines and preferences set by one's own department, institution, state, anatomical donor board, or other overseeing entities.

Further, this Guide is intended to be a living document, and as such, updated editions of this Guide will be posted on an ongoing basis to the HAPS website (www.hapsweb.org), based on input from readers and evolving practices. Suggestions are welcomed and should be submitted to the ADS Chair.

### Usage of “donor”

This Guide prefers the term “human body donor,” “anatomical donor,” or simply “donor” to refer to the bodies in anatomy laboratories used for dissection or prosection, as other choices (e.g., “cadaver” or “body”) are fundamentally objectifying. Anatomists choose the term “donor” to reflect the person's humanity and their (or their family's) decision to donate their body.^22^, ^23^, ^24^, ^25^ That said, we retain the term “cadaver” in our keywords as this language choice is not in universal practice at the time of writing.

## OVERVIEW

The rest of this Guide is divided into sections covering laboratory safety, laboratory supplies and equipment, donor preservation and biological materials care, and dissection instrumentation. Each of these is further subdivided, and for each item listed, the reader is provided with a description of that item, maintenance requirements, alternatives to selecting that item, and important considerations when considering choosing that item.

### Notes on safety and legal considerations

Any safety considerations mentioned in this Guide are based on recommendations from the collective experiences of the ADS committee members. It must be stressed that using equipment in ways other than its intended purpose is at the sole discretion of the user and that users should follow any instructions provided by the manufacturer and within boundaries set by the institution. These guidelines are developed from ADS members across the country, and guidelines vary tremendously—state to state, public versus private schools, between climate zones, and between institutions. It is critical to check with your institution for guidance and ultimate approval before making any changes.

HAPS is not responsible for injuries, exposure, equipment damage, or negligence incurred from the use of the recommendations contained in this Guide.

### Endorsement disclaimer

Descriptions provided for items in this Guide are written by the HAPS ADS Lab Resources Subcommittee members, and mention or display of these items does not constitute an endorsement of specific makes, models, or manufacturers of these items. The items displayed are solely for illustrative purposes.

Photographs included in this document were taken by the authors.

### Critical terminology and acronyms

Throughout this document, different terms to describe dissection techniques will be used. The list below includes common terms used during dissection, and are used in this Guide. Note that some terms have different colloquial usages from how they are used in dissection (e.g., “cleaning”).

Terminology Definitions

- **Approximation:** Bringing two structures together that were once separated (e.g., approximating two ends of a cut artery for suturing).
- **Button hole:** Creating a small incision in a structure, such as the skin, to use as an anchoring point with your fingers or hemostats to strengthen the grip when reflecting that tissue.
- **Cleaning:** Throughout this document, the term “cleaning” is used in multiple contexts. In terms of dissection, “cleaning” refers to the removal of surrounding tissue to isolate a structure for optimal viewing (e.g., “cleaning up the structures in the femoral triangle”). In terms of laboratory maintenance, “cleaning” is used the same as colloquially, referring to the physically washing of tools, equipment, and surfaces.
- **Dissection:** Any kind of separation or removal of tissues, regardless of instrument or method.
  ◦ **Blunt dissection:** Separation of structures without cutting. This is commonly done with fingers and probes (i.e., probing). For some, this includes using scissors in a closed state.
  ◦ **Reverse scissoring/spread scissor:** This is a modification of blunt dissection by inserting the scissors into the tissue while closed and opening the scissors to spread the tissue apart.
  ◦ **Sharp dissection:** Separation of structures by cutting, typically using a scalpel or scissors.
- **Palpation:** Feeling with your fingers.
- **Probing:** Exploring any recess with a blunt instrument (not specific to the “probe” instrument).
- **Prosection**: Whole anatomical donors, regions of anatomical donors, or organs that have been dissected in advance of viewing (such as by faculty or upper‐level students) and are ready to use for teaching.
- **Reflection:** Cutting part of a structure to be able to fold the structure back from the cut edge, such as reflecting a muscle to see deeper structures.
- **Resection:** Complete removal of whole organs or large portions of organs from the donor. For example, a “lung resection” may refer to removal of an entire lung or may refer to removal of a whole lobe of the lung, a wedge, or a segment.
- **Retraction:** Pulling back structures obscuring one's view of deeper structures without cutting them.
- **Suturing**: Technique for closing incisions or to connect structures that may have been cut or damaged during the dissection process with needle and thread.

Acronym Definitions

- **2‐PE:** 2‐phenoxyethanol
- **ADA:** Americans with Disabilities Act
- **ANSI:** American National Standards Institute
- **ADS**Anatomical Donor Stewardship Committee
- **BSL:** Biosafety Level
- **EDTA:** ethylenediaminetetraacetic acid
- **EHS:** Environmental, Health, and Safety
- **EPA:** Environmental Protection Agency
- **HAPS:** Human Anatomy and Physiology Society
- **HVAC:** Heating, Ventilation, and Air Conditioning
- **ISEA:** International Safety Equipment Association
- **NIOSH:** National Institute for Occupational Safety and Health
- **OHS:** Occupational, Health, and Safety
- **OSHA:** Occupational Safety and Health Administration
- **PEL:** Permissible Exposure Limit
- **PPE:** Personal Protective Equipment
- **PPM:** Parts Per Million
- **STEL:** Short‐Term Exposure Limit
- **USFDA:** United States Food and Drug Administration

## LABORATORY SAFETY

Safety concerns in a gross anatomy laboratory tend to focus on health issues prompted by the preservative chemicals used on the donors, injury from sharps, and potential microbial growth that can occur on the donors. Below is a list of items and practices that may help prevent such health issues or injuries in the laboratory. Formaldehyde is a known health hazard, as it can cause respiratory irritation, contact dermatitis, or irritation of the conjunctiva^26^ and has even been reported to result in squamous cell carcinoma of the nose in rats and mice when introduced to high concentrations.^27^

Though these health issues can occur in anyone, some individuals are more susceptible and will need additional considerations in the laboratory (e.g., supplementary personal protective equipment or a respirator). For example, a student with sensitive conjunctivae may require the use of goggles rather than just safety glasses. Individuals with asthma also require additional considerations as the chemicals can stimulate an asthmatic event.^26^ Onigbinde and Ajagbe^26^ recommend requiring asthmatic students to bring their inhalers into the laboratory with them, keeping an inhaler in the laboratory first aid kit, allowing/encouraging asthmatic students to take “fresh air” breaks outside of the laboratory at regular intervals, and providing training to laboratory staff on managing an asthmatic crisis.

Students or staff who are pregnant or breastfeeding also should have additional laboratory safety concerns since formaldehyde has been linked to birth malformations and even spontaneous abortions when exposed at high ambient levels.^28^ The direct effects of formaldehyde on pregnancy and birth are unclear due to inconsistent and lacking human studies on the effects of ambient levels in anatomy laboratories on pregnancy.^29^, ^30^ Despite the unknown effects, most agree that additional precautions should be taken. There is a lack of formal policies providing guidance for pregnant individuals in the laboratory^31^; however, common recommendations include wearing a filter cartridge mask^28^ or even providing alternative learning activities so that pregnant students can avoid the laboratory altogether.^31^, ^32^

Students and staff who are pregnant, or otherwise at risk for complications that may stem from formaldehyde exposure, are encouraged to consult their physicians, and laboratory staff are encouraged to accommodate any recommendations from those physicians.

### Environmental health and safety

Gross anatomy laboratories should come with a certain degree of infrastructure designed to accommodate the environmental requirements involved in hosting human donor dissection. Changing conditions over time, such as changes in traffic and occupancy, HVAC upgrades or renovations to adjacent spaces, or changes in embalming chemicals used, or changing climate, may require additional purchases or adjustments to usage of existing infrastructure. Additionally, changing overriding requirements, such as federal regulations (e.g., OSHA or EPA), state regulations, or institutional dictum, may require additional purchases to ensure the safety of faculty, staff, and students, as well as to protect the physical integrity of the human anatomy donors.

In selecting an air quality monitoring device for the laboratory, you may consult third‐party companies specializing in laboratory infrastructure and HVAC for advice. An institution's OHS/EHS department may have vendors in particular that they contract with regularly, and they may insist on taking charge of the process from selection through installation. For all matters concerning environmental health and safety, the corresponding department(s) at your own institution should be consulted before purchasing anything. Environmental monitoring devices most commonly include, but are not limited to, thermostats, humidity monitoring, and air quality monitoring.

All environmental monitoring devices should be placed at a location in your laboratory that evaluates conditions at the site where control of those conditions is most critical. For example, as high humidity can encourage microbial growth on donors, humidity sensors should be located in the immediate vicinity of your human anatomy donors. Placing the humidity sensor on the other side of the room from where any donors are held would be unhelpful. Care should also be taken that these are not affected artificially by things like airflow and environmental exposure. For example, a thermostat that is located on a wall that receives direct afternoon sun would initiate temperature changes to the rest of the (relatively cooler) room to become uncomfortable. As another example, a humidity sensor placed near a vent would show artificially low humidity levels in the room, as airflow would locally mitigate high humidity conditions.

As for maintenance, installed devices listed below should be inspected annually, at minimum. Filters should be cleaned or replaced per manufacturer specifications, or more often as needed. If additional or separate maintenance is required, it is listed alongside the device below.

In any case, trust your senses and advocate for your laboratory! If anyone detects that something is “off”—condensation forms on surfaces, the laboratory still smells strongly of chemicals, students complain about irritation, or air is sufficiently bothersome to require breaks, there is still likely a problem that is not being addressed by existing infrastructure, and either replacement or repair is required.

#### Air quality monitoring

Air quality affects the respiratory health of people working in the laboratory space, and poor air quality may encourage microbial growth. Air quality monitors are wall‐mounted devices that monitor air quality specifically for formaldehyde exposure, demonstrated as parts per million (PPM).

Alternatively, one‐time wearable devices may be purchased (Figure 1). These are small badges that are worn in the laboratory during dissection for a specified period of time and shipped out to an outside laboratory for analysis. Note that air quality may change during a semester, as donors come and go and are at varying degrees of dissection. The first day of dissection may have higher levels in the laboratory than the last day, and spikes in volatile chemical concentration may occur when body cavities are opened.

**FIGURE 1:**
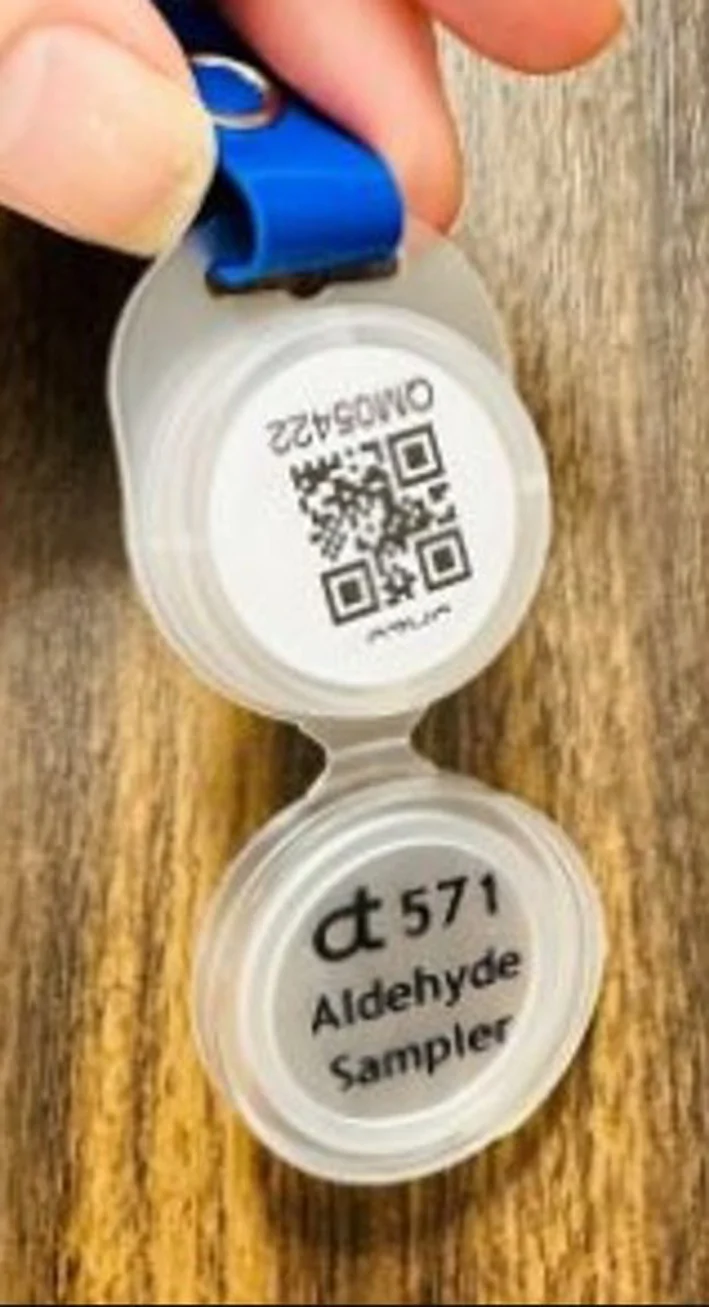
Representative picture of a wearable air quality monitoring device, which may be sent to the manufacturer for analysis.

#### Air purification

Air purification devices take in air and remove particulate count to below a specific advertised level. They may be freestanding or incorporated into a larger HVAC system. Increasing airflow may temporarily reduce high PPM but is not a permanent solution.

HVAC systems with high turnover rates may already keep levels down sufficiently that these devices are not needed, or only on an intermittent basis (e.g., when HVAC work is being done or renovations are being carried out).

#### Humidity monitoring

Excessively high humidity in your laboratory space encourages accelerated decay and microbial growth. Excessively low humidity accelerates desiccation of tissues^33^ and may irritate respiratory passages and mucus membranes of workers in that space. Humidity may fluctuate widely depending on season and local climate.

Humidity monitors are wall‐mounted devices, usually incorporated into a programmable thermostat. The sensor apparatus should be able to monitor humidity as well as automatically adjust HVAC activity to maintain a set, low humidity point. Humidity monitors can also be found as mobile, battery‐operated units, to be used in conjunction with temperature sensors and will alert the user to conditions that require manual adjustment.

#### Temperature regulation

Temperature fluctuations, particularly when the room becomes overly warm, may encourage microbial activity that causes decay. Thermostats are wall‐mounted devices, usually incorporated into a programmable thermostat, that maintain ambient room temperature to within a specific range. The sensor apparatus should be able to monitor the room temperature as well as automatically adjust HVAC activity to maintain that temperature. Alternatively, portable temperature sensors may be used, particularly in laboratories with varying conditions.

#### Respirators

Respirators are wearable face masks (full‐face or half‐face, with goggles) to prevent hazardous airborne substances from being inhaled. They are used when formaldehyde or other vapor/particle exposure exceeds permissible limits, or when students may have a lower safe or comfortable exposure threshold than the greater student population (e.g., asthma, pregnancy, strong sensitivity to odors). An NIOSH‐approved filter against formaldehyde specifically should be chosen. *They are not replaceable with single‐use surgical face masks or N‐95s*.

Importantly, respirators require users to be trained on proper use and on limitations to the one you have chosen. You should check with your institution's EHS/OHS departments for more information and required training.

Acceptable PEL for formaldehyde exposure, per OSHA, is 0.75 ppm as an 8‐h time‐weighted average, and/or a STEL of 2 ppm for 15 min of exposure.^2^, ^34^, ^35^, ^36^

*Maintenance:* Respirators require proper fitting for each wearer and require adjustment each time. They must be maintained, cleaned, and stored according to manufacturer specifications.

#### Safety binder

To ensure a safe working environment, the anatomy laboratory safety binder must be updated consistently, making sure all necessary safety documents are current and readily available. A safety binder may be made available digitally for quicker access, but your EHS department may still require that a physical copy be present in easily accessible areas in your laboratory. This process involves several key activities, primarily focused on keeping personnel informed, procedures accurate, and documentation up‐to‐date.^37^ Common pages include the following:

- **Chemical Data Management (SDS):** All Safety Data Sheets (SDS) must be immediately accessible and systematically organized, often in alphabetical order by the chemical or product name. Any outdated SDS should be removed and replaced with the most current version. The binder itself should be durable, clearly labeled, and highly visible (e.g., yellow binder) for quick identification.
- **Emergency Protocol Review:** Emergency procedures for potential incidents like fires, chemical spills, and medical emergencies must be reviewed and updated regularly. Current emergency contact information is essential. Emergency egress and evacuation routes should be clearly described and posted. Personnel training on the location and proper use of emergency response equipment (such as safety showers and eyewash stations) is mandatory.
- **Routine Equipment Checks:** Safety equipment must undergo regular inspections. For example, eyewash stations need monthly activation tests, and safety showers require quarterly flushes to ensure proper function and hygiene. All equipment, including showers and eyewashes, must be kept unobstructed and accessible. Storage cabinets and ventilation systems must also be routinely checked to confirm they are functioning correctly and are not being misused.
- **Personnel Awareness and Training:** Every person working in the laboratory must know the location of the safety binder and its contents. Regular, targeted training must be provided, covering the laboratory's unique safety procedures and the specific hazards associated with the materials being handled.
- **Documentation Verification:** All required safety records must be present and accurate. This includes logging injury/illness reports, medical consultation records, and training logs. Furthermore, all chemical containers must have clear, correct, and unambiguous labeling to prevent dangerous mix‐ups between similar‐looking substances.

### Sharps management

A dissection laboratory of any kind requires the use of sharp instrumentation to cut tissue. Proper storage, maintenance, and disposal are essential to the safety of staff, faculty, and students alike and are a basic requirement of all BSL‐2 laboratories^21^ (not just human anatomy laboratories).

#### Sharps disposal bin

Sharps containers are puncture‐resistant, self‐sealing red plastic containers with a one‐way input site that keeps sharps from spilling out when tipped. They are USFDA regulated (class 2) medical devices which must adhere to strict criteria.^38^ These should all have a biohazard label displayed prominently. Care should be taken to purchase sharps containers from a reputable source, and as they are regulated items. “Makeshift” alternatives should never be used.

Sharps containers are single‐use disposable items that must be disposed of according to your institution's biohazardous/sharps disposal protocols. Note that disposal may come at some cost to your laboratory.

#### Blade removers

Blade removers are handheld or wall‐mounted containers with a single slot for blade insertion. The user inserts the blade while still on the scalpel handle, and the flask removes the blade from the scalpel handle and deposits it directly into a sharps container which complies with sharps container requirements. The entire container is then disposed of according to the institution's sharps/biohazard disposal protocols.

Dissectors should be trained on how to use their blade removal device properly, to avoid jamming of the device or injury to the user. Gloved hands and scalpel handles should not be oily or slippery. A blade remover should be present at each location where scalpels are used.

Dissectors may be instructed on how to manually remove blades from scalpel handles instead of using these devices. If you elect to go this route, choose a method that requires students to use instruments (never their hands) for blade removal and provide a small sharps container at every location where scalpels are used.

#### Sharpeners

Instruments with blades intended for repeated use (e.g., scissors, knives) dull with use. There are companies that offer sharpening services, but they can be pricey and will require that you be without your instrument for some period of time. Owning a sharpener can allow you to restore an instrument to its original sharpness when used properly, with minimal turnaround time. They can be found as special‐use sharpening devices, or a whetstone.

As with any device, improper use can damage the sharpener or instrument, causing injury to the user. Blades should only be sharpened on an as‐needed basis. Sharpening blades on a routine basis more frequently than the blade needs will only result in excessive shaving away of metal. Serrated blades should not be sharpened in‐house without specialty training or a specially designed sharpener that can accommodate them. Finally, single‐use blades should never be sharpened.

*Maintenance:* Whetstones need to be cleaned and oiled and checked regularly for chips and other damage. Sharpeners should be inspected regularly for loose screws and other damage.

### Personal protective equipment (PPE)

In addition to the PPE that you provide based on your BSL classification and other institutional requirements, laboratory users must wear clothes covering all skin except for hands, neck, and face, and hair must be pulled back securely. You may elect to provide some additional protective materials (e.g., a protective gown with long sleeves, face shields, hair elastics) or provide a list of materials choices to let students select what they are personally most comfortable with. Lists such as this can be especially useful in regions with four discrete seasons, where what students choose to wear into school for the day can vary, and students may otherwise accidentally leave their home wearing inappropriate laboratory attire (e.g., shorts, open‐toed shoes).

Bear in mind that some dissectors may be more sensitive to laboratory hazards than others, such as those who are pregnant, have asthma, or develop severe headaches with strong odors. Those dissectors may wish to don additional PPE or take additional precautions, which should be accommodated to the greatest extent possible. Note also that some institutions may have their own guidelines for PPE use for these dissectors, so appropriate department(s) should be consulted and the laboratory should retain those guidelines for easy reference.

Single‐use PPE items will be disposed of in biohazard waste streams, and as such, their disposal may come at some additional cost to your laboratory. However, many items are universally required (e.g., gloves, safety glasses) and some items (e.g., laboratory gowns) may have reusable alternatives for daily, routine use (e.g., laboratory coats).

All fabric garments, including PPE made of fabric (if chosen), have a tendency to retain laboratory odors. To minimize this, encourage dissectors to wash all fabric after each use, ideally the same day as they are worn. Laundry detergents with lipase as an ingredient are especially effective at eliminating oil stains and reducing hydrophilic odors.^39^ Peroxide‐based additives may be added to the laundry cycle to boost cleaning power. A very small amount of ammonia may be added to the rinse cycle to strip any residue from fabric, and fabric softener can be added to help mask lingering odors. Air‐drying fabric can help prevent buildup of odors, as hot dryer cycles can “set” whatever is left on fabrics after washing.

PPE should always be stored in a “clean” area of the laboratory, immediately adjacent to the “dirty” area of the laboratory. Students should be discouraged from walking through “clean” areas of the laboratory with PPE on, even if freshly donned. For removal, dissectors should carefully consider the order in which they remove PPE to avoid self‐contamination. PPE should be kept out in an uncovered area, or in a container that is uncovered during times when students must access it. It should not be accessible from “dirty” areas and should be clearly organized in a sensible order regarding order of donning, order of sizes, and keeping mandatory items stored in a more prominent, accessible place than more optional items.

#### Disposable gloves

These provide a barrier between working hands and contaminated surfaces, including direct contact with tissue and preservation chemicals. A range of sizes should be kept in stock at all times, particularly medium and large sizes, as these tend to be the most commonly worn. Ideally, they should be 6 Mil thickness, non‐vinyl, non‐latex, examination length or greater than 12 inches in length, and have a beaded cuff. Vinyl should be avoided as it is brittle and can allow for formaldehyde penetration in less than 15 min^40^; latex should be avoided as it is a potent allergen.^41^ A beaded cuff prevents the cuff from rolling up onto itself, and examination length gloves offer waterproof protection further up the arm, and may be used to cover the ends of sleeves (which prevents wicking).

Double‐gloving is a useful practice, allowing students to change the outermost pair of gloves frequently while keeping the one next to their skin on throughout the laboratory. Nicks in gloves will often only break the outermost pair of gloves, and so dissectors' skin is more protected with this method. This also permits students to have a “clean” set of gloves for removing some PPE that is reusable or comes into close contact with more sensitive areas (e.g., head covers). Regardless of type of gloves and length of use, all gloves used in the gross anatomy laboratory are *single‐use items* and should not be reused.

#### Safety glasses and goggles

Safety glasses are plastic and feature shields which protect the eyes from all directions. They must be water‐impermeable and conform to ANSI standard Z87.1‐2020.^42^ Goggles similar to those found in chemistry laboratories and full‐face shields can also serve this purpose.

Consider supplying safety glasses designed to slip over prescription glasses. Also consider offering anti‐fog spray for the inside of glasses, goggles, or face shields, as moisture from dissectors' breath may fog them up (particularly when face masks are also worn).

*Maintenance:* These should be cleaned and sanitized after every usage. Proper care permits glasses to be worn over the course of many semesters' worth of wear, reducing cost.

#### Sleeve covers

Impervious covers for the forearms between gloved wrist and upper arms protect dissectors' laboratory coats and their skin from contact with donor fluids. They can also be used on dissectors' legs to make their attire laboratory‐appropriate if they arrive with legs exposed, though the ability to do this is dissector‐dependent. Sleeve covers typically come as one‐size‐fits all.

*Maintenance:* Sleeve covers may be water‐resistant paper or plastic, single‐use, and should be disposed of each time, or reusable fabric, and washed after each use. Waterproof materials such as those used to make sleeve covers should always be allowed to air‐dry and should never be tumble‐dried.

#### Laboratory coats

Laboratory coats are worn over a dissector's clothing to protect what they are wearing from chemical and/or tissue contamination. Wearing one, buttoned up, can help reduce odors from lingering on underlying clothes. These may feature fitted elastic cuffs that fit snugly around the wrists, but are not universally preferred. The ends of sleeves may be tucked under examination length gloves or covered with sleeve covers to prevent wicking.

Dissectors may purchase their own, in which case they should be clearly labeled and stored in a dedicated area along with other student‐owned items. Otherwise, the laboratory may purchase many and provide them as a shared resource.

*Maintenance:* Dissectors should wash their own laboratory coats as often as is practical and possible; ideally, this would be after each use. Use of fabric softener can make the fabric slightly less prone to wicking and may help mitigate penetration of laboratory odors. Laboratory‐owned gowns should also be washed as often as possible, though facilities available for that purpose will be the main driver of washing frequency.

#### Disposable aprons/single‐use gowns

Fluid‐impervious covers to be worn over a dissector's clothing can protect what they are wearing more effectively than a simple fabric laboratory coat. These are especially useful when a volume of liquid, or tissue or liquid spray (e.g., during calvarium removal), is anticipated. These should cover the entire anterior surface of the wearer.

Even if you do not intend for these to be worn for every dissection, it is a highly recommended item to keep in stock for specialty dissections. They are waterproof, and as they are disposable, require zero maintenance. Consider keeping on hand a variety of sizes for laboratory users. Bear in mind that as they are impermeable, wearers may become overheated when conditions are warm or dissections are strenuous.

#### Disposable hair covers

Disposable hair covers prevent contamination of donor tissues in the hair, and vice versa, and as such they help to maintain hygiene standards in the laboratory. They may also help reduce smells from lingering in the hair. They are typically made of lightweight, fluid‐resistant material with an elastic opening that secures around the head.

Some students may opt to supply their own surgical cap, which may be made of fabric and thus is washable. Encourage students to wash these after every use. Students who wear a head cover in their daily life may use that in place of a disposable head cover or a surgical cap, though they should be encouraged to tuck any loose ends so as to not accidentally wick up liquids.

Finally, care should be taken upon removal, and dissectors should consider the order of PPE removal to minimize self‐contamination.

#### Disposable shoe covers

These are worn to prevent contamination of tissues on shoes, maintaining hygiene standards in the dissection laboratory. They are useful for preventing tracking of chemicals and biological materials from inside the laboratory to the outside, and vice versa, helping to keep laboratory floors cleaner. They typically are made of lightweight, fluid‐resistant material with elastic openings that secure around the ankle or calf. Ensure proper fit to prevent slipping hazards, and consider providing multiple sizes. Ideally, they will have non‐slip soles.

In addition, laboratories may provide a sticky mat at the laboratory entrance or at the PPE changing area to reduce outside dirt from being tracked in and prevent tissue or other things stuck to the bottom of a shoe (if a shoe cover tears or slips off) from being tracked into the hallway. These are very effective, though they need to be replaced fairly frequently.

### First aid

Institutions may have rules governing what should be in a laboratory first aid kit or may require use of a specific kit. If this is the case, the chosen kit should conform to those standards. Keep an easily accessible guide describing what type of reporting is necessary if first aid is provided, or if a student must use mitigating infrastructure (e.g., eyewash stations).

**Call 911 for all emergencies**.

#### First aid kit

First aid kits may be offered pre‐assembled and come with a variety of supplies for the treatment of minor injuries, including cuts, which is a common injury in the anatomy laboratory. If the institution allows it, a similar one can be assembled piecemeal, or supplement a pre‐assembled kit with personally preferred additions.

Commercially prepared kits will come with some universal indicator that they are for first aid, such as a prominent red cross. Self‐assembled kits should be kept in a container that is labeled with the same universally understood signage.

Place multiple kits around the room for easy access in the event of an urgent situation. Keep one by handwashing sinks for easy cleaning and dressing of wounds. All kits should be placed low enough as to be ADA‐accessible.

*Maintenance:* Contents of kits expire on different timelines, depending on the item. Kits should be checked at minimum annually for expired items.

#### Eyewash station

Eye flushing devices are required by OSHA anyplace where there is potential for eye injury from corrosive chemicals, splashes of solutions containing 0.1% or more formaldehyde, or exposure to other hazardous materials that could harm the eyes.^43^ This may be a bottle, a sink attachment, or a permanent standalone fixture in the room, depending on needs and institutional requirements.

*Maintenance:* Bottles should come commercially prepared and expiration dates should be monitored. Discard all bottles that are expired and replace stock when it is low, but not out. Eyewash stations—both sink attachments and standalone stations—must be flushed and inspected regularly, and a log of this must be kept near the eyewash station. If water from the station has an “off” odor, does not flow at a sufficient rate, or is off‐colored, it must be taken out of service and repaired before use. *Depending on your institutional requirements, this may suspend certain activities from taking place in your laboratory in the interim*.

#### Emergency shower

Emergency showers are installed inside the laboratory itself, in an area quickly accessible and without obstruction. They are intended to be used by personnel who become contaminated during a chemical spill. Your institution will have specific requirements for when and how personnel are to use the safety shower, such as how long to be in the shower and under what circumstances and who to contact for follow‐up.

*Maintenance:* Like eyewash stations, these also must be flushed and inspected regularly, and a log of this must be kept near the eyewash station. If water from the station has an “off” odor, does not flow at a sufficient rate, or is off‐colored, it must be taken out of service and repaired before use. *Depending on your institutional requirements, this may suspend certain activities from taking place in your laboratory in the interim*.

#### Hypoglycemia and lightheadedness response

Some individuals who come into the anatomy laboratory, particularly for the first time, may become light‐headed or pass out. This can happen for a variety of reasons, including hypoglycemia, stress from interacting with donors, a vasovagal response, or locking their knees while standing for long periods of time.^44^

A common reason for hypoglycemia is when individuals choose not to eat before coming to the anatomy laboratory, as they worry, they will feel nauseous when confronted with a donor. This is non‐intuitively counterproductive. Emphasize to anatomy laboratory users that they should eat before coming into the laboratory, as this reduces their chances of feeling faint or passing out. For those experiencing hypoglycemia, it is useful to stock in a clean area of your laboratory sugary drink packets, water bottles, squeeze packets of applesauce, and similar items that are high in sugar and quickly ingested without requiring utensils. Keep these near the door or immediately outside of the laboratory, as students must not consume anything inside of the laboratory.

For those experiencing a vasovagal response or other stress response, keeping instant ice packs, smelling salts, and essential oils on hand can be useful.^45^

In all instances, stools should be kept around the room for students to sit on if they are feeling unwell for any reason. Students should always be permitted to sit down or leave the room whenever needed. Monitor students for those who look pale, withdrawn, teary, wobbly, or appear to be dissociating. Encourage students to keep an eye on one another and look out for similar signs in their peers.

*Maintenance:* All food items and volatile items (e.g., smelling salts) expire, and expiration dates must be minded. Single‐use items must be replaced when supplies are low (but not yet zero).

## LABORATORY SUPPLIES AND EQUIPMENT

### Furniture: Dissection tables, lighting, and cameras

Dissection tables are specifically designed to facilitate the dissection of human bodies, since they are designed to meet expected weight capacity and safety requirements. Various types of tables are available; however, any dissection table should be stainless steel for easy cleaning and non‐reactivity to commonly used embalming chemicals. Tables should also incorporate a drainage system, which may simply be an angled surface with a drain port, or just a drainage hole.

Many vendors who sell human dissection equipment also specialize in autopsy and pathology equipment, so you should be mindful that the two are not synonymous and are designed with different requirements in mind. For example, an “autopsy cart” or a so‐called “cadaver cart” is not usable as a dissection table, since they are intended for short‐term intermittent use, are not as stable, and do not have any means for drainage. Dissection tables are designed to house human anatomy donors on a longer‐term basis and can facilitate drainage of accumulating liquids. An “autopsy table” or “embalming table” has additional features intended for working with fresh human bodies and the biohazards that accompany them, such as an attached sink to wash blood away, which is beyond the needs of a typical dissection laboratory. Tables that are not designed for academic human dissection in mind should be avoided.

#### Standard table

Tables can be expensive, especially if accessories are also purchased. Consider current and possible future laboratory designs to help choose the right dissection table for the laboratory. Fortunately, standard tables (Figure 2A) can come with a range of features, with some being more essential than others. Standard tables are typically mobile, with lockable casters for moving around the room. Most either feature recessed or creased edging. Recessed edging prevents pooled fluids from spilling over and facilitates attachment of some bridge‐style attachments. Creased edging also prevents pooled fluids from spilling, though the boundary is shallower, so it is slightly less effective. However, it does permit bodies to be moved onto and off of the table more easily. Some other common features include adjustable height, lids, or ventilation. Specialty types of tables are discussed below.

**FIGURE 2:**
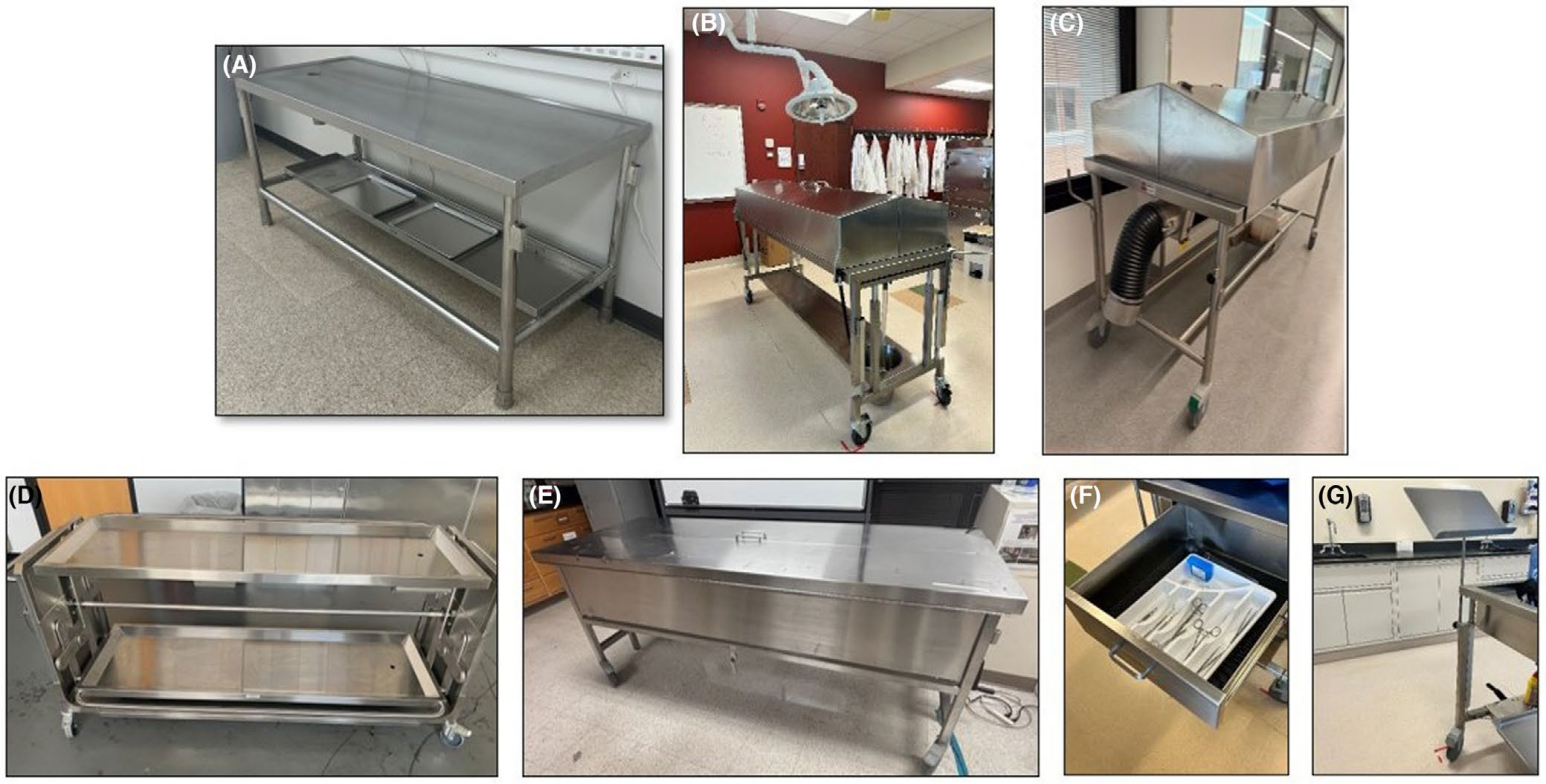
Representative pictures of different tables and table accessories. (A) Standard. (B) Clamshell. (C) Downdraft. (D) Two‐body rotating. (E) Submersion. (F) Table drawer. (G) Table stand.

*Maintenance:* Tables should be regularly inspected for secure, stable joints, that wheels are mobile and casters easily lock and unlock, that permanent accessories are still held on securely, and that all edges are free from congealed oils or tissue. Drainage outlets should be kept clear of debris or other obstructions. Tables should be cleaned with a degreasing agent frequently, with care taken to prevent any cleaning solution from coming into direct contact with the donor.

#### Clam shell table

The name of this table (Figure 2B) refers to the hinged lid that splits in the middle and folds underneath the table when opened, similar to a clam shell. When closed, it provides a complete enclosure of the table surface. This type of table enclosure may be lockable, providing additional security to your donors, and they are compatible with downdraft airflow mechanisms. But this type of table does come with some drawbacks. The lid folds under the table, which limits storage space on a lower shelf. The lid must be secured properly while under the table, or it will break free (and directly into your dissectors' shins). Additional hinges mean additional cleaning and inspection are required. Finally, they are also typically more expensive than traditional tables. You should consider both your current and future laboratory design needs when selecting table features.

*Maintenance:* In addition to maintenance required for standard tables, clamshell tables require inspection and lubrication on hinge mechanisms, and that the means of securing them under the table while in use is in good repair.

#### Downdraft table

Downdraft tables (Figure 2C) are ventilated dissection tables that pull air, odors, and chemical vapors downward and away from the faces of those around the table when connected to a negative air source. These tables are ideal for rooms that are being retrofitted to accommodate human dissection and do not have sufficient HVAC infrastructure to change over total air in the room enough to reduce formaldehyde vapor burden.

Typically, these tables feature a hollow‐frame bed that communicates with a port below the table. The bed of the table is perforated around the edges. When connected, the HVAC outflow tubing will draw air through the perforations, into the hollow space under the bed of the table, and out through the port, creating a constant downward flow of air that prevents vapors from dispersing and being inhaled. While effective, these systems can be loud, and tissue may clog perforations. These tables will not work unless the room's ventilation system has been retrofitted to permit this connection.

Dissectors should be reminded that ventilation ports and drainage ports are mutually exclusive and they need to pay attention to which is which at any given time.

Finally, since these tables physically attach to a location in the room, their location is predetermined and inflexible once established.

*Maintenance:* In addition to maintenance required for standard tables, care must be taken through the dissection process that the perforations in the table—facilitating ventilation—are never obstructed by objects such as pieces of tissue, body bags, or limbs. Oil may accumulate under the table and needs to be periodically cleaned, which may be difficult (tables' surfaces are usually not able to be disassembled). The air outflow port and all attachments should be inspected regularly to ensure that the seal between them is secure and airtight, or else the system is rendered moot.

#### Hydraulic table

A hydraulic dissection table features adjustable table elevation and tilt utilizing a hydraulic system controlled with a foot pedal. The easily adjustable elevation and tilt help with dissectors of differing heights as well as accommodating those dissectors with ADA accessibility needs.

Other tables may have adjustable heights via hand crank or manual lift and bracing of the table bed, but these features do not offer the ability to tilt the table and require the student to crouch down close to the floor to maneuver them. These tables also tend to be very heavy, and consideration should be made with regard to how often you will need to move the tables around the room.

*Maintenance:* In addition to maintenance required for standard tables, the manufacturer's guidelines must be attended to for routine maintenance and repairs. That said, hydraulics work reliably and smoothly when well maintained.

#### Two‐body rotating table

A two‐body rotating table (Figure 2D) is a dissection table that houses two human body donors, one on each of two levels. The two levels rotate so that you can alternate which donor can be dissected. Each tray has a drain pan and a drain plug. This table style permits more body donors to be utilized in laboratories with limited space, though only one donor can be accessed for dissection at a time, and both donors will rotate along the same horizontal plane, requiring clearance space beyond the table's width. Attention must be paid to ensure that discarded donor tissue remains with the correct donor the entire time. Finally, these tables may be very heavy, particularly with both donors occupying it.

*Maintenance:* In addition to maintenance required for standard tables, the rotation mechanism may need to be maintained to work smoothly.

#### Submersion table

A submersion (“dip tank”) dissection table (Figure 2E) contains a tank that is filled with a preservative solution. The bottom of the tank has perforations through the metal, so when it is raised using a hand crank or a foot‐operated hydraulic system, the fluid stays in the tank, and only the solid materials stored in the tank are raised for viewing or dissection. The tank has a lid that prevents the evaporation of fluids when the table is not in use. This table type is optimal for prolonged storage of bodies or individual parts of bodies.

Care should be taken on selecting a preservative solution as every time the donor is raised, it will degas. Students who are sensitive to chemicals may need to wear a mask. Ventilation requirements may differ from other storage methods, as this is the only table with an open container of volatile liquids. Your institution may have separate requirements for air pressure and airflow when using this type of table that do not apply to other tables.

*Maintenance:* In addition to maintenance required for standard tables, small bits of tissue may need to be retrieved from the bottom of the dip tank. Solution in the tank may need to be changed over or topped off periodically.

#### Lighting and cameras

In laboratories that were not designed originally for donor‐based dissection or where ambient lighting is fixed, overhead lighting may be insufficient for detailed anatomical work. In these settings, retrofitted supplemental lighting at donor stations can be particularly valuable and should be considered when updating existing laboratory spaces. There are a variety of lighting options to improve an anatomy laboratory. Bright LED lights within the laboratory space itself are useful, but additional lighting can help improve the dissection and/or the teaching process. Surgical lighting can include mounted or freestanding lights that are designed to not create shadows. There are a variety of styles and companies that can help. Mounted surgical lights typically involve general contractors and electricians, which may not be feasible for some laboratories. Freestanding lights come with most of the functionality of mounted lights, but can be moved and plugged into normal outlets. Some surgical lights also can include cameras within them, which can be helpful for projecting to monitors within the anatomy laboratory (e.g., to show a prosection overview or to summarize dissection steps).

Bulbs for surgical lighting can be expensive and technically complicated to replace. In addition, they may be hot to work under, especially when donned in PPE. That said, they generally provide very bright, easily focused light that is much more manipulable and creates fewer shadows than any other style of lighting.

In lieu of built‐in surgical lighting, dissectors may better illuminate specific locations by wearing a battery‐operated headlamp. Flashlights built into tablets and mobile phones may be used, though institutional and personal policies surrounding their inclusion in the laboratory in the first place may preclude this as an option.

Cameras can be used to enhance visualization of small spaces to a large group. Most commonly, cameras are used to project dissections or teaching demonstrations onto screens throughout the laboratory to aid with student visualization rather than force students to crowd around a small area and compete to see. This process is particularly useful in large spaces or when laboratory groups span multiple rooms. The camera could be a permanent installation or a mobile one, like an action camera or a tablet. Importantly, projecting these images should be part of a closed system, where the camera and the monitor(s) are connected 1:1, rather than broadcast more widely (e.g., livestreaming). As donor privacy is a paramount consideration, and breaches of donor privacy may be a violation of both ethical principles as well as legal or institutional policy, careful coordination with the overarching institution, any relevant ethics boards, and their legal team is mandatory.

*Maintenance:* Information regarding lighting maintenance differs based on brands and the model of surgical lights you have. Light bulbs will periodically need to be changed.

### Furniture: Dissection table accessories

Some types of accessories attach to the table to assist in dissections, or as storage or placement for items that are helpful to the dissector. These are available for purchase based on the type of table and are often available for purchase from the table manufacturer directly. Not all accessories are cross‐compatible with other types of table or similar tables of the same brand, so careful attention to this is critical.

Other accessories are equally helpful in facilitating dissections and in viewing prosections, but are not attached or specific to a given table. These are also listed here. All accessories should be cleaned regularly with a degreasing agent.

#### Drawers

Stainless steel drawers (Figure 2F) can be added to the dissection table at the time of purchase. Drawers are typically used to store dissection instruments at each table. Drawers can be added on the long or short sides, either under the table or under the shelf, depending on the table type. A kitchen drawer organizer can be placed in the drawers for added ability to keep instruments organized and in place when opening and closing the drawer. A drawer organizer is also easily removed for cleaning.

If your dissection table has already been purchased and does not include a drawer, instruments can instead be stored in a plastic container on the shelf under the table.

*Maintenance:* Drawer sliders should be kept lubricated and cleared of debris.

#### Under‐table shelf

By default, the space below a standard dissection table is open; however, a metal shelf can be added to allow for the storage of items utilized during dissections (e.g., books, a tissue bin, wood blocks). A tissue disposal bin dedicated to that donor may be stored there, but whole human body parts should not be stored on this shelf—they should always be kept on the surface of the table with the rest of the donor. In addition, sharps and large equipment should not be stored on this shelf.

#### Stainless steel bucket

A bucket should be placed under the table to collect the fluid drained from the donor through the drainage hole and attached tube. The bucket can sit on the floor, on the shelf below the table, hanging from a hook on the underside of the table, or a special shelf with a hole to hold the bucket can be purchased. A lid can also be purchased for the bucket. The bucket will need to be emptied periodically; check with your institution regarding how to dispose of those fluids.

#### Discarded dissection materials bin

The job of the dissector is to expose and delineate individual structures in the human body. In the process of this, tissue will inevitably be removed from the donor (e.g., meshes of nerve plexus, skin, connective tissue). Ideally, this tissue should be contained in a dedicated container at each table and not commingled with discarded tissues from other donors, though this may not be policy at all institutions. To avoid leakage, it may be lined with a bag; your institution may require that this be a red biohazard bag. At the time of cremation, this tissue should be placed with the donor and cremated along with them. Even though soft tissues effectively sublimate, families are often reassured that no tissue is truly lost or unaccounted for after final disposition.

*It is extremely important that this bin is not confused with other biohazard trash. Keep other biohazard disposal bins in a separate location from dissection materials bins. It is unethical to throw away human tissue with general biohazardous waste, and additionally, it may be a violation of your institutional or state's health codes to do so*.

#### Limb rests, book stands, and instrument trays

Leg rests are metal stands that attach to the dissection table to rest the body donor's legs on to assist in dissections of the lower limb. Book stands (Figure 2G) are metal dissection table accessories that allow you to set out a dissection manual, textbook, iPad, or other device during dissections. An instrument tray is a metal or plastic tray to set dissection instruments on when not in use during a dissection. All three of these come in two attachment forms: post‐style and bridge style.

Post‐style attachments insert into the post sockets attached to the table. As most standard dissection tables come outfitted with post sockets, this type of attachment is very commonly used. As they are simple post‐and‐socket attachments, these attachments can be moved to any corner of the table quickly and easily. Attachments can usually be secured at different heights. When secured, they can also be used as braces for limb abduction. It should be noted that limb rests in this style can only accommodate one leg at a time.

Bridge‐style attachments rest on top of the table and utilize the recessed top for stability, “bridging” over the table. They are only compatible with tables with recessed edges, but on those tables, these are very stable. They are capable of accommodating two legs at a time, or multiple books at a time, along with another item (e.g., instrument tray) on the top of the bridge. That said, they are bulky and less adaptable to changing conditions than the post‐style attachments and make regions of the donor temporarily inaccessible.

#### Head and limb bolsters

Bolsters can be used to elevate body structures of the donor to improve visibility and/or place the donor in a more accessible position for the dissector. These may be used instead of, or in addition to, limb rests. Purpose‐built head bolsters in particular are very versatile items and can be used in a horizontal (flat) or a vertical (tall) position, depending on your needs. Head bolsters can be used to elevate the sternum or lengthen the neck when placed between the scapulae, placed under the neck to elevate the cranium for calvarium removal or easier face dissection, placed under the superior brachium to facilitate dissection of the glenohumeral joint, or placed under the lower leg for foot dissection. Note that, if chosen, white will quickly discolor.

In lieu of purchasing purpose‐built bolsters, other materials can stand in to accomplish the same goals. Wood blocks work well for elevating any structure. These blocks must be pressure‐treated, as untreated blocks will grow mold and chemically treated blocks may react with embalming chemicals. Styrofoam blocks can be useful stand‐ins and are often able to be found around campus from shipment of other supplies. These should be treated as single‐use disposable items and not reused between donors (or even over the course of a full semester) as they are porous.

*Maintenance:* Aside from routine degreasing as with any accessories, these may remain in place with the donor, in their bag, between dissections. With correct care, these bolsters may last many years.

#### Limb abduction supplies

Limb abductors are used to move and hold limbs steady for access to different regions. Common supplies are waxed string, straps, bungee cords, and blocks (non‐absorbent material preferred). They may be tied to post‐style attachments to both abduct and elevate. Without these supplies, and absent those posts, students may be left holding the limbs through the duration of certain laboratory sessions.

*Maintenance:* Most of these are single‐use items. Straps may be used for longer periods of time, though they will need to be replaced periodically due to contamination with fluids and general wear and tear.

#### In‐use sharps management

While dissecting, sharps—particularly scalpels—will need to be set down between uses. Ideally, students will de‐blade scalpels whenever they are not in use; however, when taking very short breaks or switching instruments very briefly, that does not always make sense. As it is disrespectful to rest materials directly on the donor, and instruments placed on the work surface may quickly become hidden, a dedicated, obvious location to keep sharps that is separate from other instruments is useful. A dedicated tray may be used for this purpose. Better still, dissectors may bury their blades while storing them temporarily by stabbing a large, brightly colored sponge, that is kept on the dissection table but distant from active work. This means of storing bladed scalpels also enables students to pass the scalpel without risking cutting the recipient's hand.

### Furniture: Other

Students who require accommodations related to laboratory furniture or physical accessibility should consult the *Student Accommodations Handbook* provided by HAPS. This resource outlines ADA‐compliant furniture and accessibility suggestions to ensure equitable participation in all laboratory activities. Faculty should work with accessibility offices closely so that appropriate arrangements can be coordinated in advance.

#### Safety step platform/industrial step stool

A step stool is a short 1‐ or 2‐step stool to stand on to raise the dissector's height during dissection. A step stool is particularly helpful when a dissection group is composed of students of varying heights so changing the dissection table height is not helpful or possible. Students using the stool should be aware of fall risk, especially when using sharp instruments. Further, students should be reminded to watch out for stools when moving around the room. Do not purchase stools with wheels. Stools that can be stored under the dissection table and are not a trip hazard are ideal.

#### Other seating

Seating is generally limited in the gross anatomy laboratory to promote safe movement and to maintain proper body mechanics during dissection. Students should generally stand while actively working at the donor table, unless there is a need for a physical accommodation. Stools may be used for brief note‐taking, observation, or when referencing atlases or guides and must be kept clear of walkways for safety considerations.

#### Moveable trays/rolling carts

Movable, rolling trays/carts can provide a clean, flexible workspace during dissection. Students can use them to keep instruments, reference materials, and other supplies organized and within reach while maintaining a clear dissection table. Some are designed to reach over the table so that instruments can be readily accessible only when needed and out of the way when not. These are also useful for storing laboratory examination supplies so they can be brought along with the instructor while tagging structures, saving valuable time and energy. Care should be taken to keep walkways clear and to lock the wheels of the cart when in use.

### Storage for laboratory‐owned and laboratory‐supplied items

The gross anatomy laboratory is a highly variable space, but all spaces need storage for books (e.g., dissectors, atlas), dissection instruments, and tissues saved for future use. There are different commercial and common household items that can keep objects contained and isolated. Consider carefully placement of these items around the laboratory, particularly when thinking about clean versus dirty spaces and how dissectors will use them in practice. These items, like everything in the laboratory, will require periodic cleaning with degreaser.

#### Book and tablet storage

Laboratory‐kept materials such as books (e.g., dissectors, atlases) need to be stored in good condition. If you intend for your dissectors to use these materials during dissection and have some available for use when not dissecting, consider two different storage locations (one for “clean” use and one for “dirty” use). Dissectors kept in a “dirty” area for use during dissection will become greasy and should not be commingled with “clean” texts. Greasy dissectors usually cannot be cleaned but still often retain usefulness for many years. Consider laminating pages in your dissector manuals to extend their lifespan. By placing “clean” books in a covered bin or glass‐fronted bookshelf when not in use, the books may be kept free from dust, grease, or other dissection materials.

Instead of a bin, books can be kept in a dissection table drawer or on a book stand attached to the donor table (if so equipped).

Digital tablets may be used instead of paper‐based books with digital versions of dissection instructions, atlases, dissectors, or other useful reference materials. As tablets can be covered with plastic (e.g., plastic wrap, encased in a zip‐closure plastic bag), they may better avoid contamination that can come with paper texts, and the plastic exterior can be changed out periodically. Digital materials may be more easily updated and may sometimes facilitate students to take notes as they go. These are, however, much more fragile if dropped or exposed to large liquid spills.

Special consideration if using a submersible table may be needed. In this case, placing books away from the table might be best to protect them from fluids when opening and closing the table.

#### Dissection instrument storage

Laboratory‐kept materials such as dissection instruments (e.g., probes, scissors, scalpels) need to be kept at the table in good condition. A plastic bin can be placed inside the donor's bag or shell storing the instruments, or a metal, autoclavable storage bin may be used if desired. Instruments may also be stored in an organizer placed in the dissection table drawer (if equipped) or on the shelf below the dissection table. A drying rack for inside the storage bin is also useful. When not being regularly used, dissection instruments may be kept in a centrally located, accessible area to reduce clutter.

If using a submersible table, placing the instruments outside of the shell is necessary. Using a bin to keep instruments together and placing it underneath the donor can be used in this scenario. If using bags, placing instruments between the bag and table can keep instruments contained without a container.

#### Tissue buckets/bins

Buckets or other plastic containers/bins with lids can be used as storage containers for organs and other soft tissues (e.g., brains, spinal cords), both short or long term. If you plan to preserve tissues for many years, a tighter seal is needed to limit the evaporation or leakage of the preservative chemicals. If the container will be used often, damage may occur to the lid so a more durable container may be required.

*Maintenance:* These storage containers can be reused for many years, but need to be observed for tissue state, mold, or other contaminants. Plastic zip‐seal bags can also be used as an added layer of protection. Lids on containers that are opened and closed repeatedly will eventually become damaged and need to be replaced. Consider purchasing extra lids at the same time as purchasing buckets.

#### Glove storage

A wall unit to hold and organize gloves—whether they are laboratory‐provided or student‐provided, offers students and faculty easy access to gloves during the laboratory. Note the depth of the organizer compared to the glove box sizes you purchase. If the glove boxes do not fit securely in the organizer because it is deeper than the boxes, then they may be accidentally pulled out from the organizer when removing gloves.

### Storage for student‐owned supplies

Students will usually bring with them a number of items (e.g., backpack, jacket, laptop), some of which they will leave with each day, and some of which will stay in the laboratory for the duration of their course (e.g., laboratory coat, apron). To keep their things separate from institution‐owned supplies, and to keep personal items clean, storage should be offered by the laboratory that achieves this.

#### Personal item storage

Students sometimes bring bags and outdoor coats to the laboratory and need a place to store them. Depending on the laboratory, these can be stored in the hallway or in a separate room, but if this is not available, a space should be provided for this in the laboratory. Options include mounting hooks, storage cubbies, lockers, or a dedicated table. All of these options require little to no maintenance outside of occasional cleaning as needed.

#### Laboratory coat storage

A place may be provided for students to store their laboratory coats in the laboratory in the event they do not want to take them home after every class. Laboratory coats can be stored on numbered hooks installed on the wall in the laboratory so that you can assign hook numbers to students and they can more easily find their own laboratory coat at each class. If laboratory coats are provided for general use, store these separately from dissector‐owned laboratory coats.

If space and budget permit, providing lockers next to the laboratory coat hooks can make for a convenient place for students to also put their bags, umbrellas, or other personal items.

### Laboratory examination supplies

In‐laboratory gross anatomy practical examinations (“bell‐ringers,” “practicals,” “pin tests”) are usually performed using human body donors, supplemented with bones, models, or other related items. Different items can be used depending on the structure to clearly and unambiguously indicate the structure being tested. Care should be taken when using any of these, as most of them have the potential to destroy the structure when used carelessly or excessively, and that all structures on an examination should be labeled and checked for clarity prior to students' arrival.

When setting up gross anatomy examinations, consideration should be given to logistical challenges, such as workflow, student privacy, minimization of crowding around questions, and how students may write down answers. Minimization of the number of assumptions students need to make during the examination frees up students' minds to consider only the anatomy questions at hand and reduces stress that arises when they are confused about where to go or what to do, creating a more equitable testing environment. Different laboratory spaces will require different combinations of the items found below, and not all items will make sense for a given laboratory.

Consideration should also be made regarding storage of these supplies—some should be housed in “clean” areas and only touched with clean hands (e.g., clipboards and pens) and some should be housed in “dirty” areas and only touched with gloved hands (e.g., pins for structures).

Finally, students who have accommodations of any kind may require adaptation or adjustment of the workflow otherwise situated. These should be considered on a student‐to‐student basis and should be informed by ADA requirements as well as institution‐level accommodations requirements. HAPS offers an A&P Laboratory Accommodations handbook that can be referenced for ADA accessibility information.^46^

#### Labeling physical structures: Muscles and bulky structures

Larger or bulkier structures may be labeled with something directly attached to the structure in question, as the size and apparent boundaries of the structure should accommodate this easily. This is usually the easiest type of labeling to use for your students.

These structures are most frequently labeled with pins. The pin sticks directly into the structure in question and is made obvious by a flag, tag, or number. Pins can be purchased that come pre‐numbered or pre‐lettered, or with ends with special shapes so they stand out. Online suppliers, hobby, fabric, and quilting stores have an assortment that is relatively inexpensive. If you need very many or to share across many courses, T‐pins may be purchased in bulk, and those used to label structures in question can be attached to a laminated tag created by the examination administrator. Do not leave these “bare” unless it is planned to also use them to tidy up structures (e.g., pinning another overlying structure out of the way).

Adhesive materials such as tape or stickers do not tend to stay in place on donor tissues for any duration of time and may destroy underlying tissue, so direct application should be avoided.

Whatever label you chose, it may be cleaned periodically with a degreasing agent, though care should be taken that you do not poke yourself! Soaking in detergent in a colander and rinsing thoroughly can often suffice. Disposable foam cubes or magnets can be used to organize the pins after cleaning.

#### Labeling physical structures: Nerves, arteries, and veins

Other structures are more difficult to pin. Structures shaped like strands (e.g., nerves, arteries, veins, thin strappy muscles) are not easily distinguished from surrounding structures without some help, and are thin enough that putting a pin through them may destroy them. In these cases, tagging structures with string and a separate tag or colored/numbered bead may be useful. A string can be gently wrapped around the structure in question, and the tag will contain the content of the question (or the number, if students are simply to identify the structure). Gentle traction may be applied to the string to help pull the structure away from surrounding ones slightly so that it stands out better. Suture, twine, and florist's wire are common choices. If the laboratory uses vessel loops for other purposes, these can be repurposed for examination.

These are typically single‐use and will be immediately disposed of, though with lamination and care, sometimes tags can survive for many rounds of examination.

#### Labeling physical structures: Holes and openings

Holes and openings can sometimes be labeled by placing something else through them, and then tagging the newly placed thing. A probe or pipe cleaner through a bony opening in the skull, for example, with a numbered flag attached to it, often works well. The best choice will be largely determined by the size of the hole and the structural integrity of the hole—a large bony opening (e.g., foramen magnum) will have different requirements than a smaller fleshy opening (e.g., nares). Tape may be used to cover the hole for labeling, though care should be taken not to cover too much surrounding the hole (or students will be confused). In some circumstances, modeling clay can be used to fill the hole or opening in question, and then, the clay is pinned directly. Clay will need to be disposed of each time when used on a human anatomy donor.

#### Labeling physical structures: Bones and models

Bones and models are inherently more durable than fleshy human structures, but come with their own challenges. Structures cannot easily be pinned, and even if they are, the hole left by the pin would remain on the model/bone in perpetuity. In these cases, labeling tape from most scientific supply companies works very well and can be used on both bones and models. Stickers and modeling clay work well, too.

Instead of label tape, bones or models may have specific structures painted using water‐based paint. These can be applied immediately before the examination and washed off, though especially with porous structures, the structure may become permanently stained. Mistakes with paint may be hard to correct in the moment if it is realized only mid‐examination that the labeling is suboptimal. Dry erase or permanent markers may be used for more precise application, though these come with the same staining risks. Whenever paint is used, it is recommended that the same structure as the one about to be labeled is also found in the room and left unlabeled (creating a “testing” version and a “study” version).

#### Station dividers

Station dividers are physical barriers that can be placed between questions to isolate tagged structures when multiple tags are located in a tight space. Plastic folders work well, as do paper and cardboard. Note that anything porous will wick fluids and should be treated as a single‐use, disposable item. Permanent items will eventually take on odors and will be unusable for other purposes quickly.

#### Clipboards and writing instruments

Laboratory examinations are often taken on paper with numbered blanks. To provide a writing surface, clipboards can be provided for students to carry from station to station. Writing utensils can also be provided. Before deciding where to store these items, decide if they are going to always be touched with clean hands or gloved hands. If with clean hands, they should be stored away from the “dirty” area of the laboratory and writing instruments should be stored in the same place. In this case, then students may also bring their own writing implements. While taking their examination, students must be careful to not touch any structures or any other surfaces with their clipboards and pens for the duration of the examination. If part of your setup is that students may not touch structures or other things while taking their examination, keeping these as “clean” items may encourage students to keep distance from structures that they may accidentally otherwise disrupt.

Alternatively, students may take their examinations on digital tablets. When covered with zip‐shut bags, screens can still be used.

#### Traffic guidance materials

Laboratory examinations that have students following a pathway for each question can be confusing. Creating guides, such as signs or floor markings, can help remove the confusion of which question to go to next. These guides can be done with tape on the floor in the shape of arrows or by laminating directions with arrows to set on tables to guide the flow of the examination.

Anything applied to the floor can be removed by foot traffic and can become a tripping hazard. Also, some students are so absorbed in their examination that they will not recognize guides in any form. If tape has been placed permanently on your flooring (e.g., by Environmental Health and Safety), your tape should be a different color from theirs.

Instead of using guides, and if laboratory space allows, you can create an examination that has the student only traveling to the right or left to reduce confusion and remove the need for guides. Also, proctors can direct any students who lose their way in the examination.

*Maintenance:* Each examination may need new guides applied. Any guides should be removable, especially in a shared laboratory space.

### Cleaners and cleaning supplies

Most cleaning solutions are safe to use in the laboratory, though all come with specific considerations and all have the potential to damage tissue or laboratory items when used inappropriately. Care should be taken to consider features that are not explicitly reactive with laboratory chemicals, but can damage or negatively affect things that they come into contact with.

#### CAUTION: Cleaning solutions to avoid

**Some cleaning solutions—those that are chlorine‐based or peroxide‐based–—react dangerously with common laboratory chemicals, such as formaldehyde and other fixatives, or degrade tissue severely**.^35^, ^47^ These chemicals would ideally be stripped from the laboratory environment entirely, and faculty and/or staff tasked with purchasing or cleaning laboratory space should be advised not to bring in outside cleaning solutions. Examine cleaning solution labels closely for these features or ingredients before purchase or use. If these types of cleaners must be in the laboratory, they should be stored far from all cleaners that may be used anywhere near human anatomy donors and labeled clearly.

#### Cleaning supplies—safe for laboratory use

The process of cleaning items used in the laboratory can be made easier with solutions that can be found in many household kitchens. These are listed here as well.

For all cleaning supplies, expiration dates should be minded, though most have a very long shelf life. Expiration dates on cleaning supplies and solutions sometimes do not speak to the quality of the product inside as much as the integrity of the container over time—so be sure to also check for leaks and eroded seals.

#### Detergents

Detergents emulsify fats to allow for breakup of fatty residues. Some may contain enzymes to assist with digestion of unwanted molecules. However, they usually also come with other cleaning agents, so check to ensure that no chlorine‐ or peroxide‐based ingredients are listed.

Detergents can be good for cleaning instruments, stainless steel, and oil spots in clothing. Do not allow detergents—with or without enzymes—to come into contact with the donor. Rinse detergent thoroughly after use and wipe dry to remove any remaining traces of detergent. Avoid detergents with moisturizers (often advertised as “leaves hands soft”) as these will leave a hazy residue on your surfaces that may be difficult to wash off later.

#### Caustics

These high‐pH cleaning agents are powerful degreasers and good at cutting caked‐on grime. Do not store these near acids.

This type of cleaning agent is very effective at removing caked‐on, sticky, oily residue, especially if the residue is old. However, it is severely caustic to tissue—living and otherwise—and so gloves should always be worn while using it and close attention needs to be paid that it does not come into contact with the donor. Rinse this very thoroughly after using it. Spray degreasers coupled with an abrasive pad can work, though this will require more work.

#### Concentrated enzymatic cleaners

These solutions contain high levels of enzymes, usually proteases and lipases, to break down biological materials (adhered to non‐porous materials). These are excellent for soaking instruments or other stainless steel or non‐porous objects with significant accumulation. Results may be mixed with porous materials, and enzymes may not fully rinse out from the interior of porous materials. These cleaners should not come into contact with donors at all, as they will erode tissue significantly. All instruments cleaned with enzymatic cleaners should be rinsed very thoroughly.

#### Melamine erasers

These single‐use products work like very fine‐grit sandpaper to abrade away thick and/or greasy surface contamination. These are also great for caked‐on grime and will not react with donor tissues, but will abrade away underlying surfaces if used inappropriately.

#### Drainers and strainers

This can include a plug for your sink drain and colanders for soaking instruments and rinsing them en masse. These require periodic washing with a scrub brush and cleaner (detergents work well) to remove caked‐on oils and tissue, as the holes can become plugged with tissue and oily residues can build up over time. To avoid this, colanders can be lined with a cheesecloth or similar disposable fabric.

#### Sponges and brushes

Sponges and brushes should be purchased with several basic considerations in mind. They should be durable and designed to not hang on to tissue irretrievably. Second, they should not scratch delicate dissection instruments or stainless steel tables. Tables and instruments that are etched (e.g., from steel wool) will accumulate debris and oils and may quickly become uncleanable. You should provide a variety of sizes, and they should be stored wherever cleaning solutions and sinks are.

## DONOR PRESERVATION, MATERIALS, AND BIOLOGICAL MATERIALS CARE

### Donor tracking systems

Donor tracking is critical to anatomy laboratory management. Every donor and donor‐derived tissue should be traceable from the time of receipt through final disposition. Each donor should be assigned a unique identifier upon arrival in the laboratory. This identifier should remain associated with the donor through all preparation, storage, teaching, research, and disposition activities. Personal identifying information should be stored separately from operational records and accessed only by authorized personnel.

A consistent identifier convention should be adopted. Identifiers may incorporate information such as year of receipt, donor sequence number, or an institutional coding system. Whichever is selected, it should be simple, scalable, and intuitive.

The tracking system should maintain a complete record for each donor. Information such as donor identifier, date received, preservation method, current storage location (and history of storage locations), preparation status, course or program assignments, tissues used from the donor, and final disposition information should be maintained and saved such that old information is difficult to impossible to delete. Other information from the donation form that may also be recorded and tracked will vary based on what information the donor program collects and discloses. For example, disclosed information could include cause of death, medical history, occupation, or even the full name of the donor. Electronic databases are preferred for ease of use and ease of access. Routine audits should be performed to verify that donor inventory matches the records.

Tracking systems serve not only operational needs but also donor stewardship. Accurate records demonstrate respect for donors by ensuring that all donated remains are accounted for throughout the process in education and in research.

### Biohazard waste disposal

Biohazard waste is an inevitable aspect of laboratory use and care. All personnel who have access to the anatomy laboratory should be trained and have proof of training at Biosafety Level 2 (BSL‐2). Appropriate disposal of biohazard waste should be in accordance with institutional Environmental Health and Safety (EHS) regulations.

**Extremely important:** Many laboratories will use some kind of lined bin at each table for storage of tissue removed from each donor (tissue that has been “cleaned” from structures). These containers should be separate from the biohazard waste stream and dealt with according to Institutional, State, or Anatomical Gifts Board protocols. *These containers must be obviously, visibly different and physically removed from the biohazard waste stream to avoid student and EHS staff confusion*.

#### Biohazard waste bags

Biohazard waste bags should be available in abundance in the laboratory, lining rigid containers. The container may itself be disposable (e.g., large cardboard box). Biohazard bags are often used for the disposal of human tissue, but these bags should be separate from waste receptacles and should be associated with each donor individually. Make sure to consult with the managers of your body donor program for the preferred use and separation of hazard waste and donor material. Sharps go in their own sharp containers (which are already labeled for biohazardous waste).

Bags should be replaced when they are ¾ full. Consult with your institution's EHS team for scheduling of biohazard waste pickup. Disposal of biohazard waste may be an additional expense for your laboratory.

#### Biohazard waste bins

Bins for disposing of biohazardous waste, such as full and sealed sharps containers, used PPE, and paper towels used to sop up spills, should be abundant and placed conveniently for dissectors. Dissectors should not have to walk far to reach any given biohazard waste bin. Each bin should be lined with a red biohazard bag, which will be discarded and replaced as needed. The bin itself may look like a regular large garbage can and may or may not be on wheels or may be itself a disposable cardboard box, which would be taken out along with the biohazard bag for removal.

Biohazard waste bins should be clearly identified as such and be easily distinguished from non‐biohazardous waste bins and from discarded human tissue disposal bins. Disposal of all items in the laboratory must go through the correct waste stream.

### Wetting solutions and antimicrobials

This section assumes that the laboratory uses formaldehyde as its major fixative during embalming, as it is by far the most common chemical at the time of writing. Other embalming solutions, especially formaldehyde‐free solutions, will require very different care that is unique to the fixative and your laboratory's needs. This is addressed in *Discussion* here broadly, but the manufacturer and/or preparer of your embalming fixative typically provides specific instructions, which should be consulted.

A small amount of water may help “clean” structures of debris, but does not serve as a humectant (a solution that facilitates moisturization) and will not help to remove microbial contamination of tissues. In fact, letting structures remain overly wet with solutions that do not have active humectant or antimicrobial properties, or diluting solutions that do have those properties, may encourage growth of mold and bacteria.

Wetting solutions have humectant properties that help tissues remain supple and resilient despite long‐term environmental exposure. Humectants work by drawing moisture by binding environmental moisture and bringing it into drier areas.^48^ Antimicrobial solutions prevent, inhibit, or slow down fungal and bacterial growth on tissues.^49^ These should be used frequently and liberally, and it may be useful to set a timer in the laboratory to remind students to do this before time gets away from everybody.

Wetting and antimicrobial solutions may be purchased separately and used separately, may be purchased and mixed in‐house, or may be found in commercially prepared solutions that come pre‐prepared with both properties. Consideration to potential reactivity or degradation of solutions should be made when mixing in‐house. Storage and waste stream disposal of both types of solution should be governed by your institution's EHS requirements (e.g., formaldehyde may need to be stored in a fireproof locker). Both types of solutions may degrade over time, so expiration dates should be clearly marked as well, though they generally have a very long shelf life. Oftentimes, expiration dates do not speak to the quality of the product inside as much as the integrity of the container over time—so be sure to also check for leaks and eroded seals. Finally, as humectants work by drawing moisture from moister areas into drier ones, they may be counterproductive in laboratories with very dry conditions.

Examination of the donor for excessive desiccation or microbial growth, inside and out, should be part of students' and dissectors' routines.

#### Pre‐mixed wetting solutions

Several different commercially available humectants can be used to maintain tissue moisture and pliability. The best product for any given laboratory should be discussed with the body donor program's embalmers. Refer to the label on the product and consult your embalmer regarding best use of the product you purchase.

#### Humectant: Glycerol

Glycerol is a common humectant that gives tissue a moist, oily texture.^50^ In small amounts, especially when mixed with other humectants, glycerol preserves the pliability of tissue well.^48^ Glycerol and glycerin are the same chemical compound, though typically use of the word “glycerin” refers to a lower‐quality grade product and “glycerol” refers to a product with much higher purity and quality assurance.

Glycerol will only offer its humectant properties for a short period of time, so repeated application is necessary. Too high of a concentration of glycerol will leech fluid away from tissues, so when testing out mixtures, a low concentration should be used initially and gradually titrated higher. The final concentration of glycerol will depend heavily on the quality of the embalming on your donor, and the environmental conditions in the laboratory, with laboratories with very dry air tolerating far less glycerol than those with less dry conditions. Similarly, if donors are near a heat source or are in a sunbeam, their drying may accelerate with excessive glycerol use as well.

#### Humectant: 2‐phenoxyethanol (“2‐PE”)

2‐PE is a stock chemical that can be purchased from most chemical companies. This chemical is often found in cosmetics.^51^ In recent years, a diluted concentration of 5%–20% has been used as a humectant for donors. 2‐PE should be sprayed liberally every 30 to 60 min while donor bags or tanks are open. When used frequently, the solution can keep donor tissue viable for years. Unlike glycerol or fabric softener, 2‐PE will not dry out tissue unless the concentration is too low. It is common to mix 2‐PE with glycerol for its antifungal effect in the first bottle of the semester. Use a higher concentration at the beginning of a semester; subsequent bottle refills can use lower concentrations. Subsequent refills can contain 2‐PE on its own.

#### Humectant: Fabric softener

Fabric softener is also effective in hydrating preserved tissues so is an inexpensive alternative to glycerol.^52^, ^53^ However, its use can leave a color residue on the donor tissue based on the type of fabric softener. Fabric softener is otherwise used similarly to glycerol.

Some brands have a stronger smell than others, which may be effective at masking other laboratory odors but may also further irritate students who dislike strong odors.

#### Fixative and antimicrobial: Formalin (10%)

Formalin solution of 10% is the most common embalming concentration for perfusion or topical fixation. While embalming involves flowing fixatives through the vasculature or by submersion, the same solutions can be kept on‐hand in spray bottles for topical touch‐ups of regions that are showing signs of poorer fixation. Topical spray mixes can contain humectants as well.

If organs are going to be stored for lengthy periods of time in formalin, for example, in a pathology museum or a teaching collection, buffering of the 10% formalin solution is recommended as formalin is slightly acidic.

Formalin should be stored in an acid safety cabinet, and away from oxidizers, reducing agents, and metals. Its location should otherwise be cool, dry, and well‐ventilated. Environmental and safety regulations at your institution should be consulted.

Formalin is very volatile and can quickly irritate eyes and nasal passageways.^35^ Care should be used when working with the chemical, as formalin is a known carcinogen.

### Alternative embalming solutions

Some laboratories have elected to employ non‐formaldehyde‐based embalming solutions. This choice may come out of concerns about:

- **Smell:** The smell of non‐formaldehyde embalming solutions is typically mild to non‐existent.
- **Environmental disposal concerns:** Non‐formaldehyde embalming solutions may be—but are not necessarily—more easily biodegradable and less toxic to air, soil, and water systems. Depending on the solution and the volume, they may not require a dedicated separate waste stream for any disposal needs.
- **Lifelike anatomical quality:** Many non‐formaldehyde embalming solutions are more akin to “soft‐prep” fixations (below) and preserve the texture and pliability of tissues. Commonly, joints remain completely mobile. Coloration may be preserved, to varying degrees, depending on specific solution and methods used. This does, however, often result in more fragile tissues which are inherently more prone to damage than firmer fixation methods.
- **Final disposition preferences:** Final disposition for human anatomy donors is nearly always cremation, due to cemetery requirements surrounding formaldehyde limits for burial. Non‐formaldehyde embalming solutions, when biodegradable, may open up final disposition options to donors' families such as traditional burial or natural organic reduction. In any case, a family viewing after dissection is not feasible.
- **Ventilation/older building design standards:** Non‐formaldehyde embalming solutions are often not as readily volatile and therefore have reduced HVAC and air quality control requirements. For older buildings that cannot be easily upgraded, or spaces with poor ventilation, a non‐formaldehyde embalming solution may be preferable.

Popular alternatives largely involve use of low‐ or non‐formaldehyde solutions. Low‐formaldehyde solutions are generally referred to as “soft prep” or “soft fix” embalming, and non‐formaldehyde solutions are generally referred to by their proprietary name or the major component of the solution (e.g., “ethanol‐embalming”).^54^, ^55^, ^56^ Note that as formaldehyde is a powerful antimicrobial agent and fixative, non‐ or low‐formaldehyde embalming solutions may not prevent tissue decay of, or microbial growth on, your donors as well, and will require additional attention and care to prevent this.

- **Phenol:** This is often a component of formaldehyde‐based embalming solutions, to varying degrees based on embalmer preferences and the individual donor's embalming needs. It is a potent antifungal agent and disinfectant but comes with toxic exposure risks and poses a fire safety hazard.
- **Soft‐fix:** The most common form of soft‐fixation is Thiel embalming, which was developed in response to personnel concerns regarding formaldehyde exposure. This does use formaldehyde, but in very low concentrations. This solution can also be introduced after some formaldehyde preparations to rehydrate and restore pliability to stiff donors. This method is excellent at preserving donors for training students and residents at delicate surgical procedures, such as vascular procedures, otherwise unachievable with formaldehyde donors. Despite major advantages, this method requires a high degree of skill and knowledge by embalming staff and requires specialized equipment, creating an overall expensive embalming preparation. These donors also require more frequent intervention to prevent decay, consuming substantially more time from dissection laboratory staff.
- **Non‐formaldehyde:** The most commonly used commercially used solutions are usually trialdehyde‐based (e.g., glutaraldehyde) or ethanol based.^54^
  ◦ **Glutaraldehyde:** Embalming quality is similar in rigidity and duration. Glutaraldehyde exhibits many of the same cytotoxic and carcinogenic hazards as formaldehyde, though with reduced risks and higher exposure thresholds. Though glutaraldehyde exhibits lower vapor pressure at sea level, fewer perceptible fumes are emitted from the donor. Glutaraldehyde is a strong sensitizer that can trigger asthmatic and allergic reactions such as dermatitis.
  ◦ **Ethanol based:** These may be bought as proprietary solutions or may be mixed in‐house, for example, soap‐and‐ethanol based solutions,^57^ ethanol‐glycerin solutions^58^ but typically offer superior pliability and a fresher appearance. Students' interaction with these tissues is a more realistic proxy for interaction with living human tissue. Donors embalmed with these solutions may require decay prevention measures that are very different from those embalmed with other means, though being less caustic, these rarely require any kind of infrastructural enhancements for accommodation (e.g., HVAC upgrades). These donors do tend to release more fluid over time, so drainage at the table level will need to be monitored closely.

Ultimately, the choice of embalming lies with the body donation program and/or the embalmer, and what they are willing and able to use for embalming, and their choice will dictate the environmental health standards that your laboratory must meet. It is possible, however, to advocate for practices that are best for laboratory personnel and would match the preferences of all dissectors. If further resources are needed on exploring options and weighing the pros and cons of different methods, the ADS Lab Resources subcommittee can provide those.

#### Spray bottles

Common cleaning or industrial‐style spray bottles are used for maintenance, wetting solutions, and topical fixatives. Pressure sprayers can also come in handy, though these are trickier to use without damaging tissues.

Spray bottles should be clearly marked with their contents and intended purpose on an immediately visible spot and ideally a spot where hands do not routinely come into contact with it. Labels on spray bottles frequently become loose or rub off over time with exposure to spray solutions and oily, gloved hands. Consider using a truly permanent marker (one that requires Xylene for removal) covered by a stuck‐on sheet of clear plastic to reduce this. *Having chemical bottles such as these spray bottles around the laboratory, unlabeled, is to most departments a serious safety violation so close attention should be paid to this*.

### Drapes and absorbents

In addition to the bag or tank cover to keep the donor and tissue physically contained, drapes can help prevent the donor from drying, and absorbents can soak up excess embalming fluid or fluids that accumulate over time. Some absorbents may be closed up with the donor in between dissections to catch whatever accumulates over time, but these should be checked regularly as lingering soaked fabrics/paper may encourage microbial growth. In fact, many commercially available “household” absorbents (e.g., paper towels) may come with their own bioburden (microorganismal contamination), rendering their use inappropriate in any context.

#### Plastic sheeting

Plastic sheeting or bags can be used to cover donors in order to prevent drying. Bags can be used on limbs. The type, transparency, and thickness of sheeting can be variable. The ventilation of the anatomy laboratory determines whether plastic sheets are necessary or even the right type of covering. Plastic sheeting should not be used with downdraft tables, as it negates the purpose of the downdraft table. Plastic should be disposed of when torn or after each donor's time in the laboratory is complete.

#### Fabric sheeting

Fabrics (bed sheets, towels, cheesecloth) are another viable solution that are washable between uses across donors and can be soaked in humectant and/or fixative for draping between uses. This type of sheeting can be used with downdraft tables, provided that the fabric is tucked snugly on donor tissues and not obstructing ventilation perforations. They should be discarded with any signs whatsoever of mold or mildew, but otherwise can be reused perpetually. Do not wash or dry these fabrics in commercial washing machines, as these are not sterile and will deposit bioburden onto the fabrics.

#### Other absorbents

Sponges can be kept at donor stations to absorb accumulating fluids, but should not be stored in the bag with the donor. Commercial sponges often contain bioburden that may then be transferred to the donor. If they are used, they should also be visibly different from the sponges used for sharps storage.

Chucks—absorbent pads with an impermeable backing—can also be placed with the donor. As these are usually medical‐grade, they are typically free from inherent bioburden and very absorbent. They should be disposed of whenever they become saturated.

### Body bags and donor containment

Donors should not be left out on tables exposed to the environment between uses. Not only does it encourage accumulation of bioburden and tissue desiccation, it is also intensely disrespectful to the human anatomy donor. The donor should be stored securely, with humanistic considerations (e.g., donor dignity, donor safety) in mind.

#### Body bags

These keep and store human donor remains while on the table, to reduce exposure of the donor to the environment, as well as preserve privacy and dignity to the donor. These are frequently envelope style with two zipper pulls, and 12 Mil thickness recommended. They may feature pulls/straps on the sides of the bag to facilitate moving the bag on the table or from one table to another, but are not strictly speaking necessary. Colors for these bags vary—different colors may be chosen based on laboratory needs.

*Maintenance:* To ensure proper drainage, a hole may be cut near the foot of the table, between the donor's calves, and align that hole with the drainage port on the table. The hole may be kept open and the bag held in place with a ring fitted to the size of the hole (though this will need to be custom‐made). When transporting the donor, the hole must be closed up first.

#### Submersion tanks

These are a specialty type of table, wherein the donor is held submerged in fixative solution and raised for viewing and/or dissecting, and is described above under “tables.” That said, they may also be used as long‐term storage for donors between courses, at which time they are removed and transferred to a body bag. These tables are heavy and bulky, and the volume of volatile chemicals may be expensive.

## DISSECTION INSTRUMENTATION

Dissection instruments are indispensable for human anatomy dissection. Some common household stand‐in supplies can work for specialty reasons, but there is no household item that replaces, for example, a scalpel. That said, they are often more expensive than purchasing household items and sometimes need to be purchased from specialty surgical suppliers. As a terminology note, items used for human anatomy dissection and surgery are referred to as “instruments,” not “tools,” and sometimes go by different names than the ones we use colloquially (e.g., “tweezers” are for home use, “forceps” are for dissection).

Dissection instruments should be thoughtfully maintained and stored to reduce overall costs and to protect student safety. They should be cleaned after each use by, at a minimum, rinsing with water. Enzymes and detergents can better degrease instruments and dissolve away tissues stuck in hard‐to‐reach joints and flanges. Instruments should be dried thoroughly and stored as “open” as possible to help evaporation inside tighter spaces. For example, scissors should be air‐dried with blades open so the blade surfaces can dry thoroughly. Rusted instruments should be discarded when the levels of rust are sufficient that they no longer work or cannot be cleaned well.

Occasionally, instruments can be obtained from surgical facilities. Expired suture works perfectly fine in an anatomy laboratory, though it cannot be used in surgery. Clamps that are slightly bent or past a pre‐prescribed lifespan may also be unsuitable for surgery, but fine in the anatomy laboratory. These can be very low‐to‐no cost ways of obtaining dissection instruments and supplies, though the availability of these will vary tremendously between laboratories and locations.

### Daily/single‐use instruments

The following instruments are suggested for a basic “dissection kit.” Pre‐assembled kits can be purchased for students individually, or these instruments may be purchased for the laboratory as a whole and shared between students and courses. Single‐use items (e.g., scalpel blades, vessel loops) should be purchased by the laboratory and not left to student discretion, unless laboratory protocols are sufficiently detailed that an exact quantity of these items can be known.

#### Scalpel handles and scalpel blades

Scalpel handles attach to disposable scalpel blades to facilitate the cutting of tissue. They feature a handle portion, as well as a blade‐attachment portion, which usually features a metal flange for securing the blade. The handle also features a small angled shelf that orients the blade correctly. There are multiple handle sizes, which only fit specific blade sizes. The handles are labeled with their size number, and usually, the side with the size number listed is the side onto which the blade is applied.

Dissection blades (aka, surgical blades, or scalpel blades) come in varying sizes and materials. They are typically packaged in sterile foils that have an interlocking mechanism which is specific to different sizes of dissection handles. These are single‐use items that should never be reused or sharpened. They come extremely sharp but dull quickly, and should be changed out frequently during dissection—the only thing a dull blade is good for cutting is your fingers! The most common handles and blade sizes (Figure 3) are as follows:

- **#3 handle:** This is the smaller of the two handle sizes and fits blade sizes #10, #11, #12, and #15. These blades are better suited for cutting delicate or very small structures and are useful in very detailed dissections.
  ◦ **#10 blade:** General‐purpose blade for making incisions and dissecting tissues.
  ◦ **#11 blade:** A pointed blade for making precise incisions and for very fine dissections.
  ◦ **#15 blade:** A small, curved blade for delicate dissections and making small incisions.
- **#4 handle:** This is the larger of the two handle sizes, fitting blade sizes #20, #21, #22, and #23. This is often the better “starter” size handle for dissectors as it is slightly larger and thus easier to wield.
  ◦ **#20 blade:** A larger version of the #10 blade, useful for larger dissections and for thicker tissues. This blade size is often the best size for skinning and removing/opening fascia.
  ◦ **#22 blade:** A broad blade for cutting through particularly tough tissues.

**FIGURE 3:**
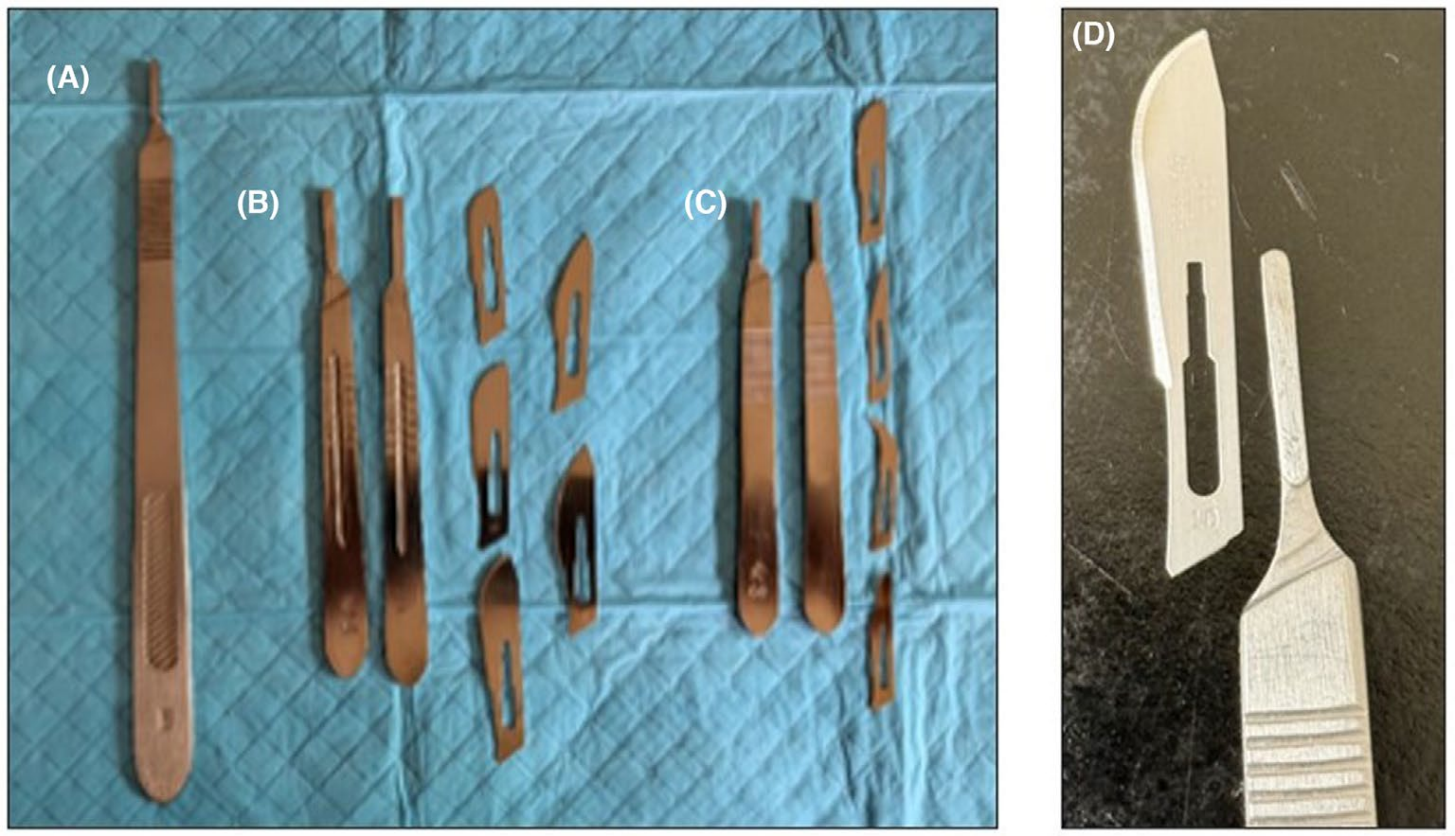
Representative pictures of different scalpel handles and blades. (A) Extended scalpel handle. (B) #4 standard handle with blades (#20, 21, 22, 23, 25). (C) #3 standard handle with blades (#10, 11, 12, 15). (D) Close‐up of size #3 scalpel handle showing flange where the #10 blade beside it would rest when attached.

*Maintenance:* Craft blades, or disposable scalpels, come with pre‐attached blades and are single‐use only. Do not reuse these, attempt to sharpen them, or attempt to remove the blade. For reusable handles, the flange may accumulate dirt and oils, which can be cleaned out with a degreaser or detergent and a toothbrush.

#### Probes

The angular semi‐blunt probe is a versatile dissection tool featuring a slender, curved shaft with a rounded, semi‐blunt tip. Its angled design allows for precise navigation through anatomical structures while minimizing the risk of puncturing or tearing delicate tissues. It is useful for identifying, separating, or tracing soft tissue planes, vessels, and nerves, and for scraping or blunt dissection. Any non‐porous blunt instrument, or closed blunt forceps, will work for this purpose as well, though the dedicated dissection probe is so versatile that it is strongly recommended to have at least a few of these around. Examples of some different probes and forceps can be seen in Figure 4A.

**FIGURE 4:**
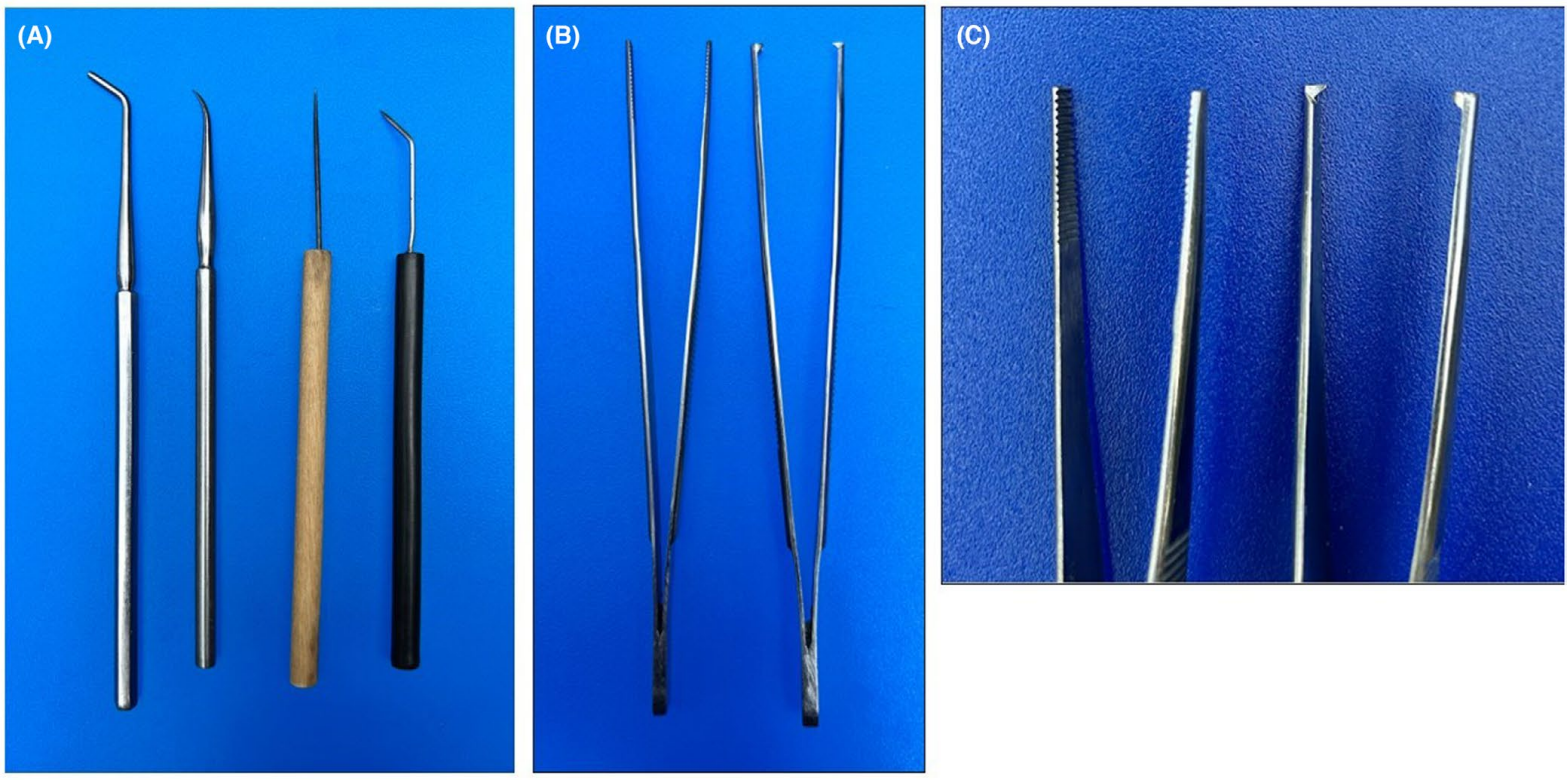
Representative pictures of different probes and forceps in dissection. (A) A variety of probes (left to right: blunt angled, sharp curved, sharp straight, sharp angled). (B) Forceps without teeth (left) and rat‐tooth forceps (right). (C) Close‐up of forceps jaws without teeth (left) and with rat teeth (right).

#### Forceps

Blunt edge forceps (Figure 4B,C) have smooth, flat gripping surfaces with rounded/blunt edges, designed to handle tissues with minimal trauma. These instruments provide a gentler grip, making them ideal for holding delicate structures or for use in areas where tissue integrity must be preserved (e.g., small nerves, arteries, veins). When the blunt edge is closed, it can also be used as a probe for blunt dissection.

Forceps can also be used to prop structures up for visibility. Finally, care should be taken when holding these for extended periods, as hands and wrists can become unnecessarily fatigued—consider using hemostats for longer uses.

Serrated (“tissue,” “rat‐tooth”) forceps (Figure 4B,C) are designed for securely grasping, holding, and manipulating tissues during donor dissection procedures. These forceps feature interlocking serrations along the inner surface of the tips, providing stable handling of delicate or slippery tissues without excessive pressure.

Typically, forceps are used heavily during skinning or when creating tension on a structure by grasping. Forceps can also be used to prop structures up for visibility. Excessive or inappropriate use of rat‐tooth forceps can easily puncture or shred structures, so these should be used judiciously. Finally, care should be taken when holding these for extended periods, as hands and wrists can become unnecessarily fatigued—consider using clamps for longer uses.

Alternatives to using rat‐tooth forceps are hemostats or blunt forceps. Blunt forceps may (or may not) grab as securely or may slip. Hemostats may crush tissue but hold very securely. Rat‐tooth forceps may shred tissue with repeated use.

Over time, the serrated edge may dull or break off. Dispose of these in a sharp container.

#### Scissors

Dissection scissors are precision cutting instruments designed to cut tissues, fascia, and vessels with control and accuracy. They typically feature two sharp blades with either blunt or pointed tips and are available in straight or curved configurations to accommodate various dissection needs. Examples of some different scissors can be seen in Figure 5.

**FIGURE 5:**
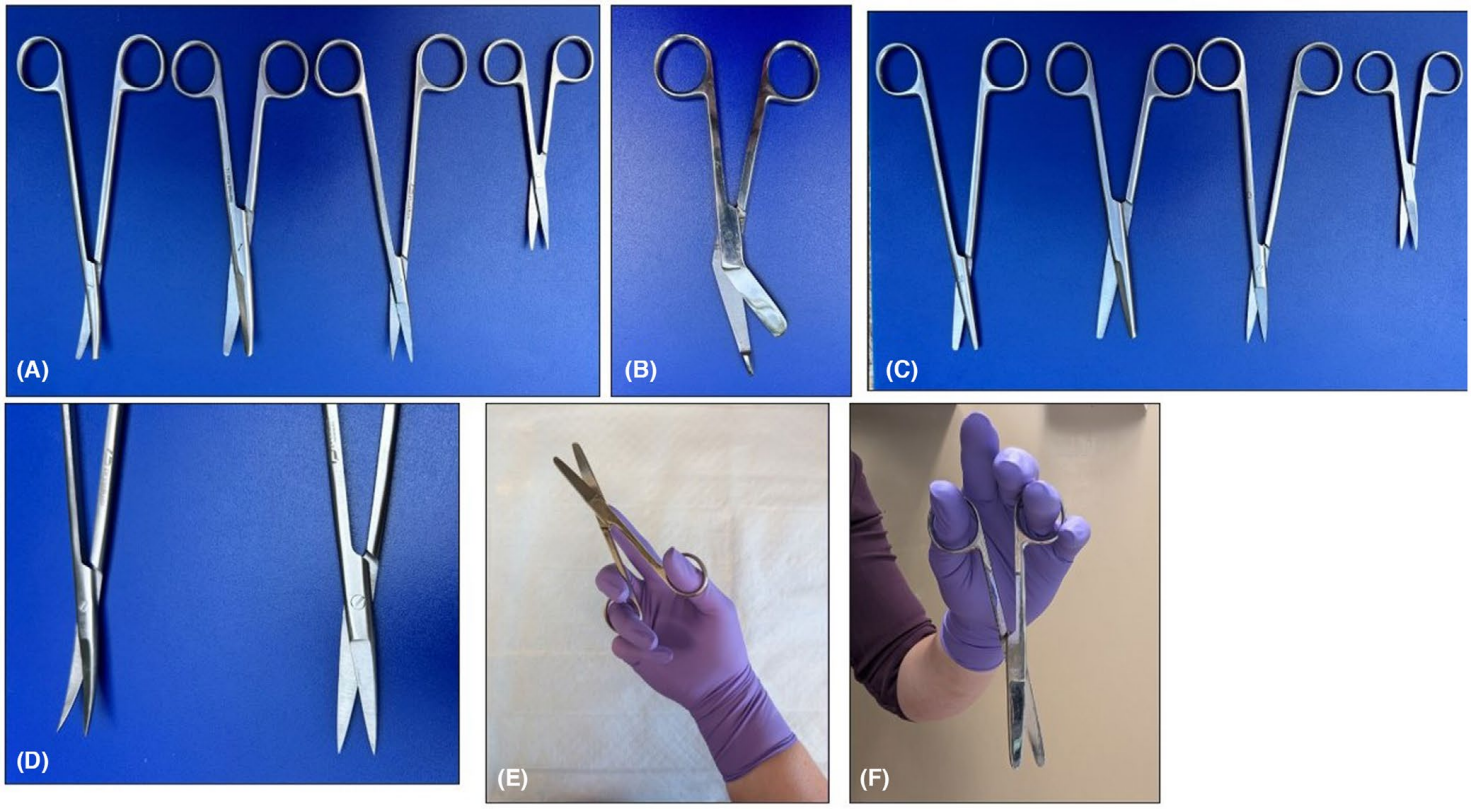
Scissors. (A) Curved scissors (left to right: Metzenbaum, Mayo, Kelly, Iris). (B) Bandage scissors. (C) Straight scissors (left to right: Metzenbaum, Mayo, Kelly, Iris). (D) Close‐up of curved tip (left) and straight tip (right). (E) Tripod grip. (F) Backhand grip.

When the sharp edge is closed, they can also be used as a probe for blunt dissection. They can also be used for reverse scissoring, in which the blade starts closed and then is opened to spread apart fascia in order to locate structures within.

Specific types of dissection scissors commonly used in donor dissections are listed below. It is recommended in a dissection kit to have two types of scissors: Iris and Metzenbaum.

- **Metzenbaum aka “Metz”:** may be curved or straight; these features long handles with short, sharp, pointed‐tip blades, good for cutting cranial nerves during brain removals.
- **Mayo scissors:** Feature one blunt‐tipped blade and one sharp‐tipped blade.
- **Kelly scissors:** Similar to Metzenbaum but shorter and sharper‐tipped.
- **Iris scissors:** Small and pointed, ideal for delicate or tightly packed locations as well as for reverse scissoring.
- **Bandage scissors:** Have one sharp blade and one flat/dull blade. The dull blade is in contact with tissue, and the sharp blade is used to cut. Useful for cutting structures overlying much more delicate structures.

In all cases, ergonomic use of scissors is essential. For ordinary, forward‐direction use, your thumb and ring fingers go through the loops. Your index finger (forefinger) rests on the box‐lock (joint) for precise control (“Tripod grip”; Figure 5E). For dissection in a deeply oriented direction, your thumb and forefinger can go through the loops, with the bulk of the handles resting against your palm (“Backhand grip”; Figure 5F).

#### Hemostats and clamps

Hemostats (also referred to as “hemostatic clamp” or “clamps”) feature a scissor‐like design with ratcheted locking handles and serrated jaws for a secure grip that can be curved or straight. When retracting for long periods of time, clamps can be applied to tissue, locked at the jaws, and the finger loop may be attached (hooked or tied) to another structure to hold it in place.

They are intended to be used to hold tissue, secure tissues in place, or manipulate small structures. They are useful for skinning and creating tension on skin. When the blunt edge is closed, it can also be used as a probe for blunt dissection. Examples of some different hemostats can be seen in Figure 6.

**FIGURE 6:**
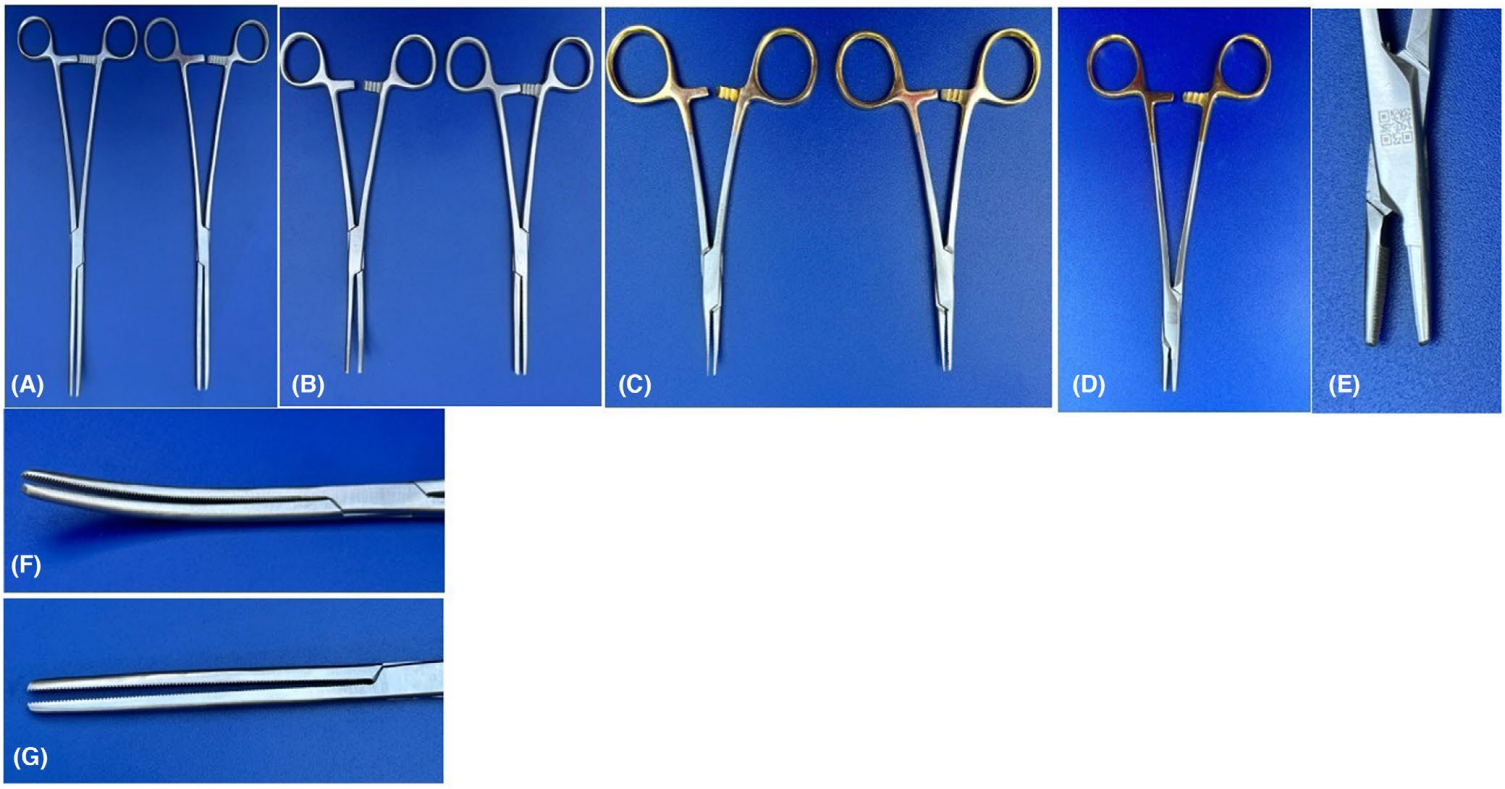
Hemostats and needle driver. (A) Rochester hemostats (left: Curved, right: Straight). (B) Kelly hemostats (left: Curved, right: Straight). (C) Mosquito hemostats (left: Curved, right: Straight). (D) Needle driver. (E) Close‐up of needle driver jaw. (F) Close‐up of curved hemostat jaw. (G) Close‐up of straight hemostat jaw.

Common types of clamps include the following:

- **Rochester clamps (**Figure 6A **):** These are typically much larger, ranging from appx 20–50 cm in length. They feature cross‐hatched grooves in their jaw for crushing tissue and a firm grip on very tough tissue (e.g., fascia).
- **Kelly clamps (**Figure 6B **):** These “workhorse” clamps are medium‐sized (14 cm long, 7 cm jaws) clamps with serrated jaws that can be an effective clamp for most tissue.
- **Mosquito clamps (**Figure 6C **):** These have short, grooved tips for handling delicate structures with precision (e.g., eyelids, small blood vessels)

Clamp tips come in multiple shapes (Figure 6F,G):

- **Straight:** Feature straight jaws that are most easily used for most applications.
- **Curved:** Feature curved jaws that can facilitate clamping into tighter spots, over more delicate structures, or with more precise control. Curved clamps can be used to remove scalpel blades safely, when the user is trained properly.

As with scissors, ergonomic use of clamps is essential. For ordinary, forward‐direction use, the thumb and ring fingers go through the loops. The forefinger rests on the box‐lock (joint) for precise control (“Tripod grip”). When possible and practical, the clamps should be actively secured using the locking jaws near the finger loops, and clamps manipulated using the muscles of the arm. Actively holding clamps shut for extended periods of time fatigues the hand excessively and exacerbates or may create repetitive stress issues.

#### Needle drivers

Needle drivers (Figure 6D) have short, blunt, grooved tips (Figure 6E) designed for precise control of a needle held between the clamp jaws. Other types of clamps should never be used as needle drivers.

Instructors may use different methods for suturing, and some methods require that dissectors handle suture needles manually. This practice must be avoided wherever possible, as it is a serious sharps risk. Puncture wounds are harder to clean and are more likely to become infected. The needle driver may be used to “tag” the suture needle whenever not in use—it should be kept attached so the location of the needle stands out. Needle drivers may be used to pop off wedged‐on pop‐off needles from suture and used to carry the needle to the sharp container.

#### Suture

Suture (Figure 7) is thread made especially for surgical purposes. Suture may or may not come packaged pre‐wedged with a needle, which may need to be cut off or may pop off with some tension. The thickness of suture is determined by gauges, with the higher the number indicating a thinner diameter. Suture can be made from different materials, including prolene, silk, vinyl, and kit gut. Dissectors may use suture to re‐attach broken structures, to secure skin flaps prior to flipping or moving donors, or for clinical practice.

**FIGURE 7:**
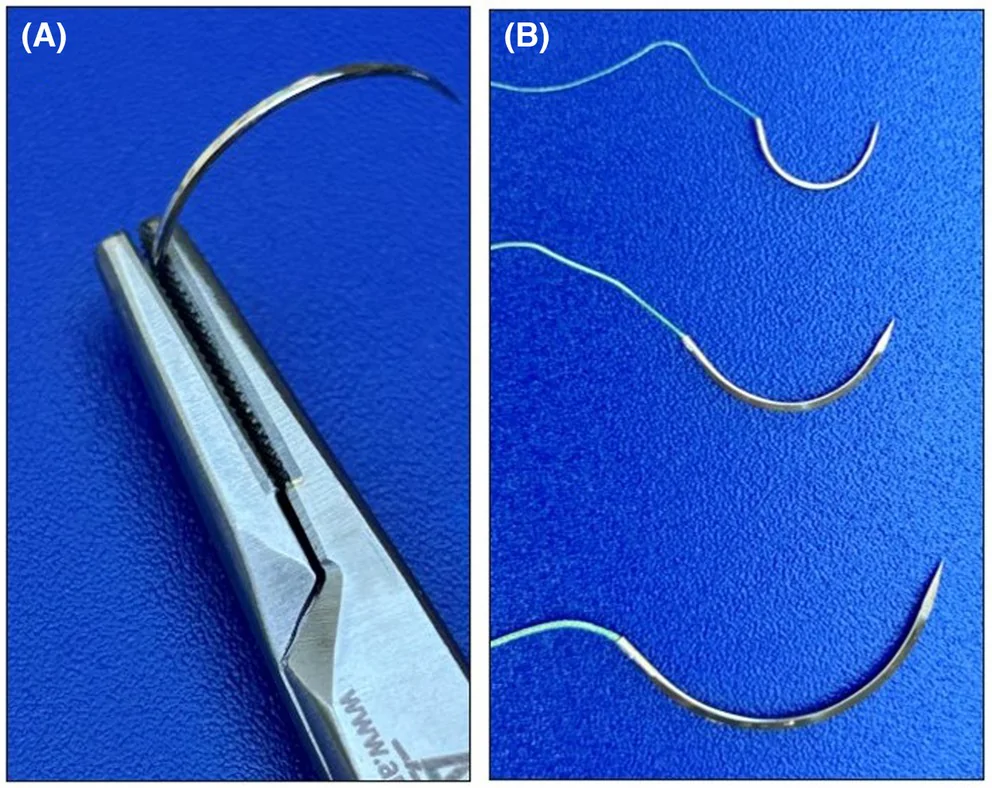
(A) Needle held by needle driver. (B) Different gauges (6–0 top, 4–0 middle, 2–0 bottom) of suture swedged on to cutting needles.

Different types and gauges of suture are indicated for different tensile strengths and wound‐healing needs of tissue. Choose thicker suture for more robust tissues (e.g., fascia) and thinner suture for delicate structures (e.g., muscular arteries). Absorbable sutures may not be recommended for very long‐term use, as they may degrade with time. Suture and suture needles should only be handled with hemostats—never with fingers.

Attention must be paid to the type of needle attached to the suture: needles come in blunt, taper, or cutting varieties, each with distinct purpose.

- **Blunt needles:** Best for friable tissues and simple tying together of structures, these feature a blunt tip with no sharp sides whatsoever.
- **Taper needles:** Feature a sharp tip but smooth sides to help penetrate tissue initially but without further cutting tissue along the way.
- **Cutting needles:** Have sharp edges to help penetrate through tougher and/or thicker tissue.

#### Vessel loops

Vessel loops are thin, usually colorful, soft silicone bands that can wrap around delicate structures like blood vessels or nerves for identification, or very gentle retraction. They may be used as a sling for these structures and tied to adjacent structures to keep them out of the way. They may also be used to tag structures for identification purposes (e.g., laboratory practical). Though these are gentle, pliable bands, they can still tear delicate structures if too much tension is applied to them.

#### Surgical adhesives

Surgical adhesives bind tissue together. This is usually to repair an accidentally cut or torn structure during the course of dissection. Surgical glue will adhere two surfaces directly to one another, whereas surgical tape will indirectly adhere them together.

You have to get surgical glue right on the first attempt—it is very difficult to re‐do a gluing application. Surgical glue may give off an unpleasant smell. Surgical adhesive tape may permit multiple attempts, but may not hold as well. Tape can also be applied around structures as a form of label.

Suture, vessel loops, or other ligature can also secure two structures together but may not be appropriate for every instance in which surgical adhesive is useful.

Finally, cyanoacrylate can be purchased through surgical or biological supply vendors, but cyanoacrylate is the same as standard hardware store Super Glue, and pricier alternatives rarely (if ever) provide additional benefit. Attention should be paid when selecting hardware store glue though that it does not contain other ingredients that make it less suitable for tissue use (e.g., craft glue, wood glue). If your institution has a forensics department, they may already have cyanoacrylate in large quantities, as this can be vaporized to reveal latent fingerprints. If cyanoacrylate is to be utilized in the laboratory, it is also prudent to stock a solvent for it (such as acetone) in case it needs to be removed from instruments or skin.

### Laboratory‐shared/specialty instruments

Instruments in this section are useful for more specialized dissections and may not be included in individual dissection kits. These do not need to be purchased in similar quantities as the daily use or single‐use items and can be shared between groups.

As for all things, laboratory instruments may experience wear and tear, especially those that are used with force applied (e.g., mallet, chisel) or mechanical (e.g., reciprocating saw). Inspection of these items for dulled edges, loose parts, insecure connections, and other related deterioration should be a frequently visited part of the laboratory's routine.

Unlike daily use dissection instruments, more specialty‐ or infrequent‐use items can be purchased easily at a hardware store without worrying as much about a dip in quality.

#### Chisel and mallet

Chisels are used with a hammer (Figure 8A) or mallet to break hard tissue, as a lever to separate hard tissue, or to scrape tissue away from bone. They are also useful for scraping erector spinae away from the spine to perform a laminectomy, or to remove bone fragments or pieces after scoring with a saw (e.g., mandible removal). Mallets (Figure 8A) are used to apply force to a chisel to break hard tissues. Rubber mallets or surgical (bone) mallets both work for this purpose.

**FIGURE 8:**
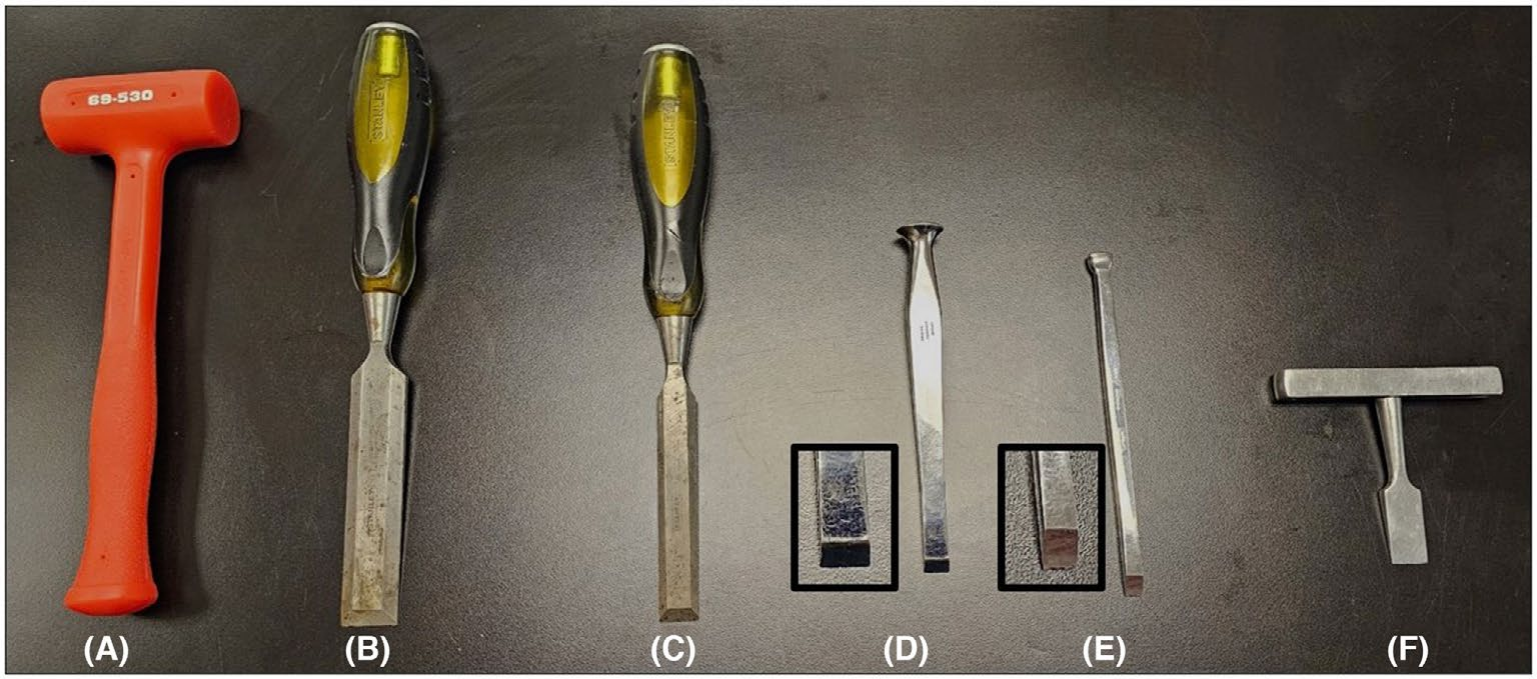
Hammer and chisels. (A) Mallet. (B) Wide wood chisel. (C) Narrow wood chisel. (D) Wide bone chisel. (E) narrow bone chisel. (F) T‐chisel.

Woodworking chisels often work well for nearly all dissection laboratory purposes (Figure 8B–E). T‐chisels (Figure 8F) feature a short chisel blade with a horizontal handle that can be hit with the mallet and can be gripped securely to pry structures apart (e.g., calvarium removal).

Probes can be used as a chisel to break very small and delicate pieces of bone, such as the orbital bone, or during sphenopalatine dissections, but this should be carefully judged on a case‐by‐case basis.

*Maintenance:* Keep these clean and degreased, as with all other instruments. The chisel may be periodically sharpened along with other sharps (e.g., scissors) when dull.

#### Retractors

These instruments (Figure 9) are used to hold back tissues, organs, or skin to provide clear visibility and access to the underlying anatomical structures during dissection. They can be used to open body cavities or spaces to mimic procedures (e.g., thoracotomy, sternotomy) or other smaller spaces. Retractors come in various shapes and sizes, including handheld and self‐retaining styles, and may have blunt or sharp ends depending on the type of tissue being retracted. Common types of retractors include the following:

- **Weitlaner:** A self‐retaining instrument with two parallel pronged blades and a ratchet locking mechanism, used to hold soft tissues apart during surgeries. It frees the user's hands by remaining in place once positioned, allowing for easy adjustment and stability.
- **Senn‐Miller:** Used for retracting soft tissues to provide better visibility and access. It typically features a single blade with multiple prongs on one end, which can be blunt or sharp depending on the application, and a single flat blade on the other end. Senn‐Millers are commonly used in small surgical sites because of its simplicity and ease of use.
- **Rib spreaders (also known as thoracic retractors):** Specialized surgical instruments used to separate the ribs during thoracic surgeries. They typically consist of two arms that can be adjusted to widen the space between the ribs, providing the user with unobstructed access to the chest cavity.

**FIGURE 9:**
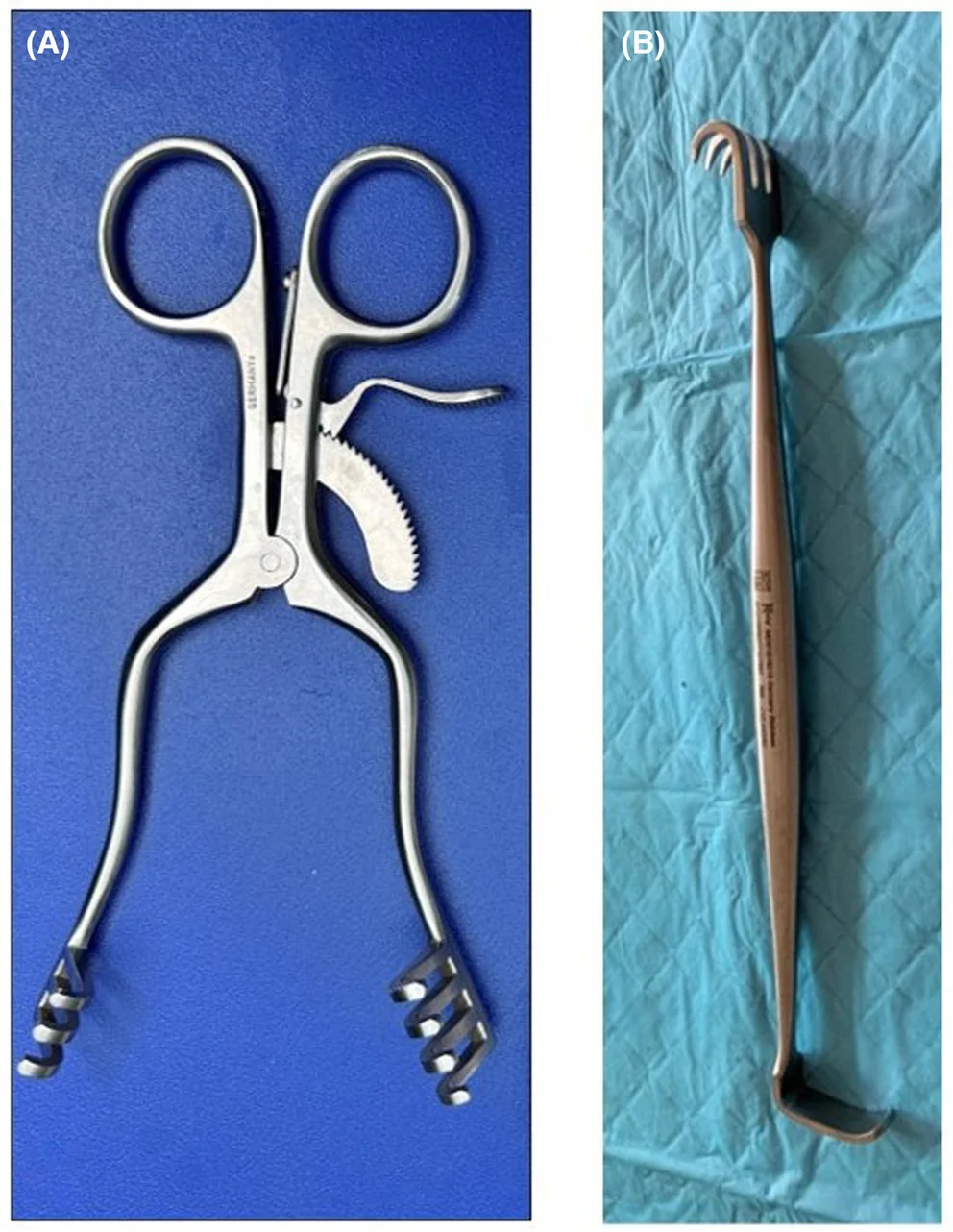
Retractors. (A) Weitlaner. (B) Senn‐Miller retractor, an example of a small handheld retractor.

Examples of handheld and self‐retaining retractors can be seen in Figure 9.

#### Rongeurs

These instruments (Figure 10) are designed to cut and remove small pieces of bone or tough tissue during dissection. They feature strong, scoop‐shaped jaws with a sharp cutting edge and are operated by a spring‐loaded, plier‐like handle. They are useful for exposing bony structures or clearing pathways for deeper anatomical exploration. They are especially useful for (indeed, designed for) removal of bone in discrete “bites.” These come in many sizes, depending on the dissection's needs.

**FIGURE 10:**
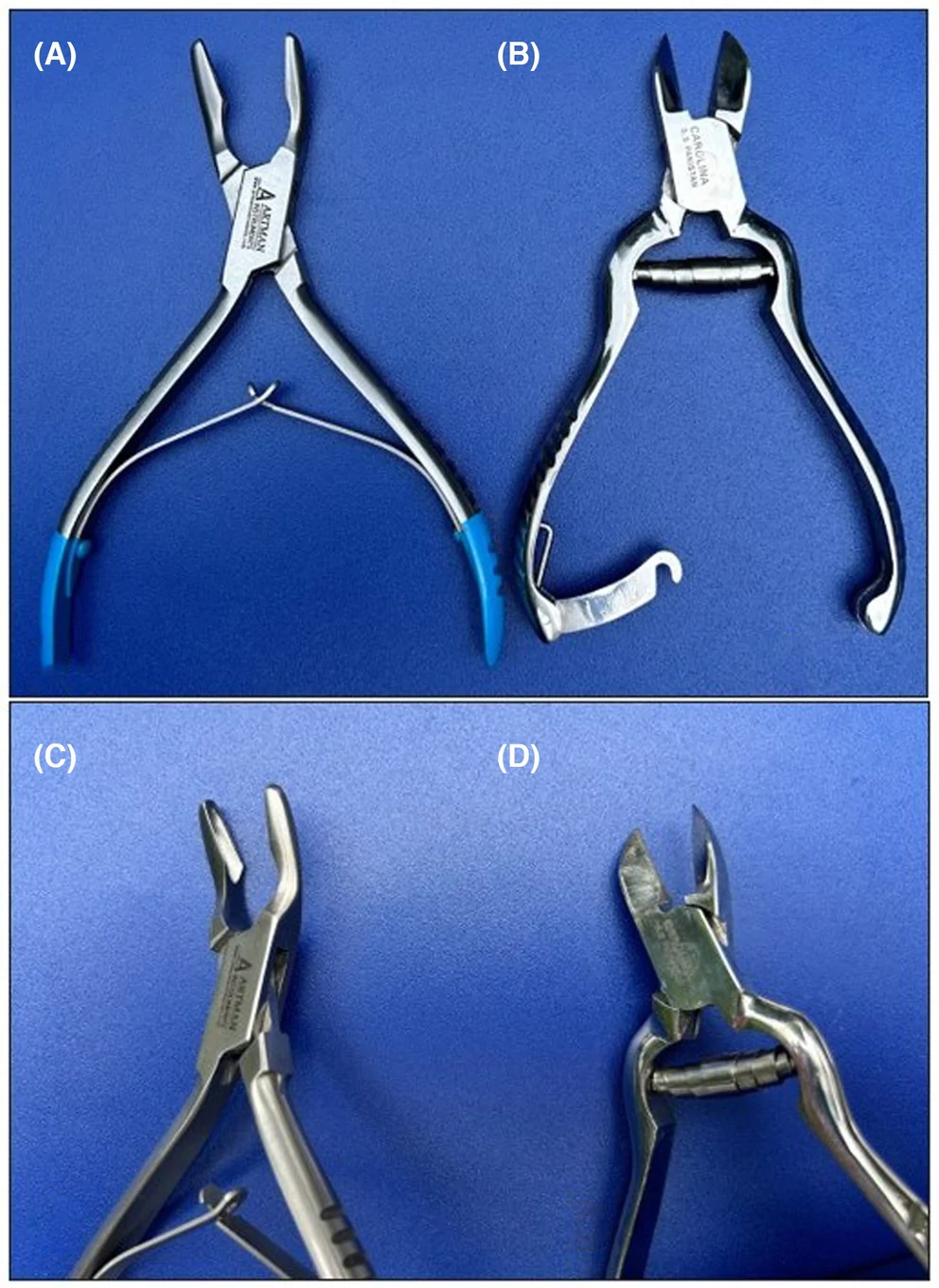
Rongeurs and Rib cutters. (A) Rongeurs. (B) Rib cutters. (C) Close‐up of rongeurs tip. (D) Close‐up of rib cutters tip.

#### Bone shears and rib cutters

Robust cutting instruments that resemble scissors but are larger, more durable, and often feature asymmetrical blades for increased mechanical leverage and cutting strength (Figure 10). They are designed for slicing through tough or thick materials such as cartilage, ligaments, fascia, and occasionally bone during dissection.

In particular, for cutting ribs, curved pruning shears from your local garden supply store usually work exceptionally well and come at a fraction of the cost. An example can be seen in Figure 10B,D.

#### Extended scalpel handle

These instruments (Figure 3A) serve the same purpose as traditional scalpel handles, and fit the same blade sizes, though they are extra long for increased reach into deep cavities during dissection, such as cutting the brainstem during brain removal. Care should be taken when using them, as they are a greater cutting hazard than shorter handles, in which dissectors exert better control.

#### Virchow knife

A Virchow knife (Figure 11) is a long, thin, double‐bladed knife used for sectioning soft tissue. It looks like a cross between a kitchen knife and a frosting spatula. They can be used to section brains or other visceral organs into axial, coronal, or sagittal sections. A very large chef's knife or butcher knife can serve this purpose equally well, though ideally the handle of the knife will not be porous.

**FIGURE 11:**
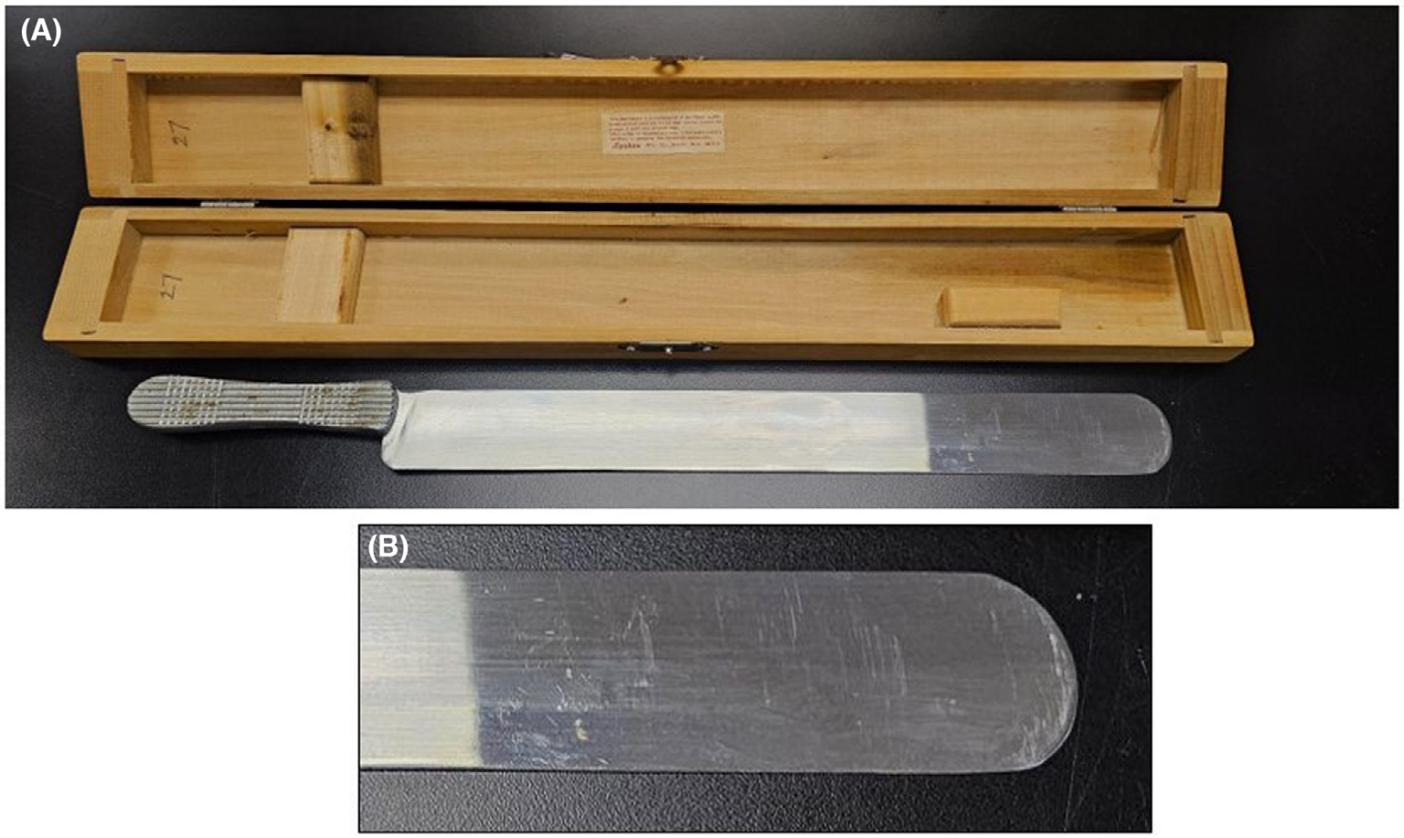
Virchow knife (A) and a zoomed in view of the blade (B).

#### Saws (handheld)

Handheld saws (Figure 12A–E) can be used to cut through all hard tissues, such as bone and cartilage. Soft tissues can occasionally be cut with a saw, but may shred tissue more than actual cutting (so discretion must be used when employing this method).

**FIGURE 12:**
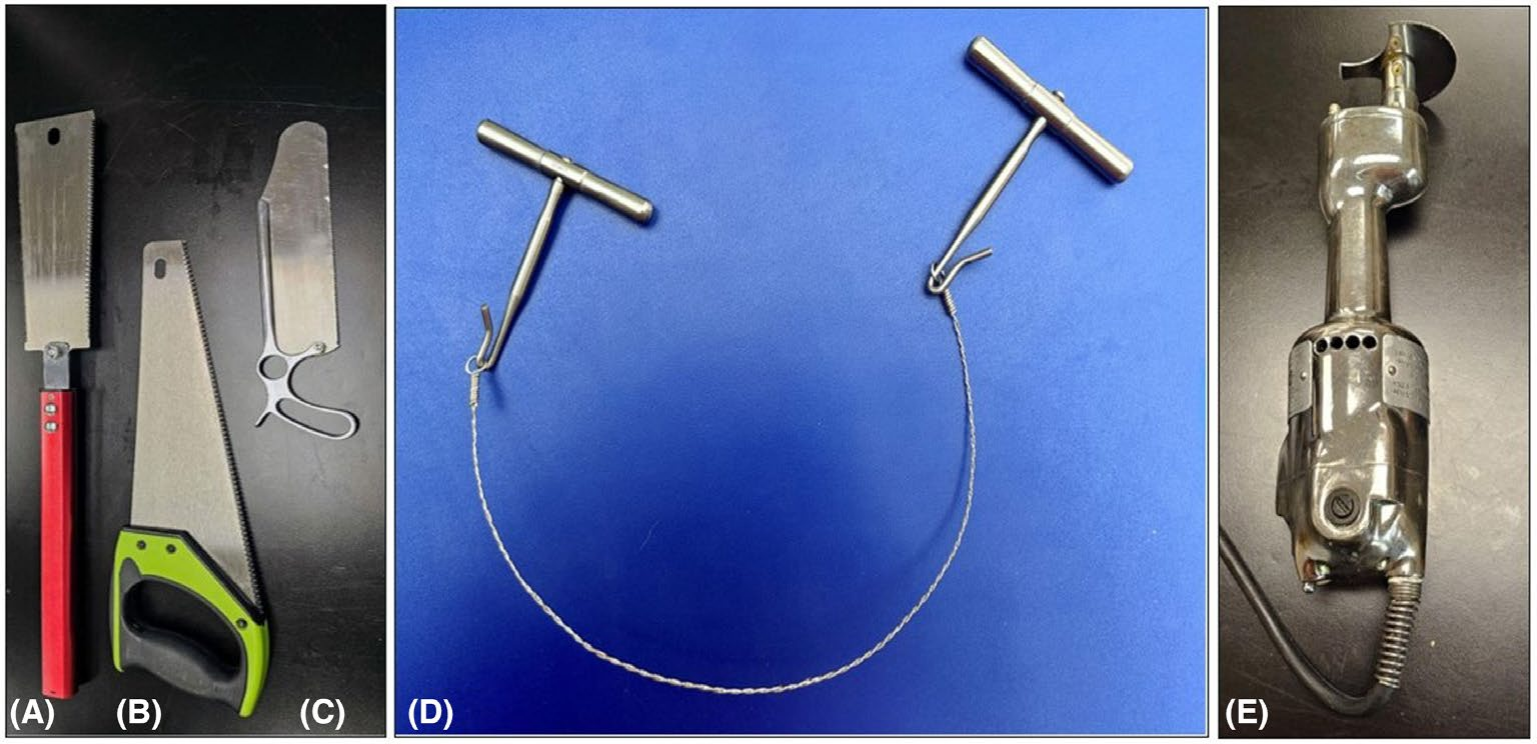
Types of saws. (A) Japanese woodworking saw. (B) Wood saw. (C) Traditional bone saw. (D) Gigli saw. (E) Electric oscillating saw.

These are excellent for creating large cross‐sections/axial sections into the head, neck, pelvis, and for removing limbs. Some, but not all, saws have replaceable blades or blades that can be re‐sharpened, extending their lifespan in your laboratory. Varieties to choose from include the following:

- **Japanese woodworking saws:** The blades are on each side so they can cut in both directions and are useful on smaller cross‐sections as the blades can be too flexible for thicker bone
- **Wood saw:** 18″ recommended
- **Traditional bone saw**
- **Hacksaw** (not pictured)
- **Gigli saw:** This amounts to a handle with a sharp metal thread that is “sawed” across surfaces to cut. They are flexible and particularly useful when structures beneath what you are cutting need to assuredly remain intact.

#### Saws (automatic/electric)

Electric saws (Figure 12E) serve the same basic purpose as manually powered saws, but are more useful in some cases and require less physical exertion in all cases. However, closer attention must be paid to maintenance—poorly maintained electric saws become extremely dangerous quickly. Manufacturer instructions for cleaning and repairing, as well as blade replacement, should be strictly observed.

Electric saws are useful for scoring bone (e.g., skull prior to chiseling during a craniectomy). They may also be used to cut through bones such as ribs and vertebrae, yet note that electric saws do not always cut through soft tissue.

Varieties of saws include:

- **Oscillating saw:** Recommended size ½–1 in. oscillating replaceable blades. Before cutting bone, clean soft tissue away as the blade may slip and cause a safety hazard. Take caution when cleaning with liquids
- **Angle grinder:** Cuts through metal
- **4.5″ circular saw:** Also useful for sectioning
- **9″ or 10″ band saw (18″ recommended if budget allows):** Different blade lengths and blade types that can be used to cut through bone or metal
- **Dremel saw:** Domes with multiple bits for cutting and grinding
- **Revolving blade autopsy saw**

#### Ceiling‐level extension cord

While not strictly speaking a dissection instrument, ceiling‐level extension cords are an exceptionally useful addition to a redesigned laboratory when using an electric saw. This is a retractable extension cord installed in the ceiling near dissection tables that provides easy access to plug in electric bone saws and allows personnel to use electric‐powered tools without the trip hazard of having a cord on the ground or extending out from the wall. These can be stationary or come attached to a sled on casters on the ceiling to extend the safe range of this cord. Take care that the resting height of the extension cord is low enough that dissectors can reach it without requiring a step stool or reaching over things that may roll out from under it.

#### Scoops and syringes

These are used to remove excess fluid and loose tissue from cavities. For liquids, scoops and syringes both works, though sopping methods for liquids (e.g., chucks or sponges) may be preferable, depending on volume. Alternatives include basters, dinner spoons, and ladles; though, consider using non‐culinary items when practical and possible for donor dignity.

## DISCUSSION

### Custom solutions and repurposing

The gross anatomy laboratory often contains specialized equipment that is both costly to acquire and may not meet the needs of an individual course or a particular space. Some instructors create their own custom solutions using materials found incidentally.

#### Retail stores and in‐house resources

Specialty vendors for anatomy laboratory or surgical supplies may sell materials that can be very high quality, but may also be very expensive and the quality level may be over and beyond the needs of a given anatomy laboratory. For example, bone shears can be purchased from a vendor who manufactures surgical supplies. These will be made to withstand exposure to blood, very strong cleaning materials, and repeated autoclaving with no warping or structural changes, and repeated machine sharpening over a very long lifespan. As materials in the anatomy laboratory do not need to be sterilized, and blade sharpening can be expensive, this may be excessive. However, many equivalent materials can be picked up inexpensively at hardware stores or craft stores—in this case, for example, garden pruning shears work at least as well as bone shears and are a fraction of the cost.

Hardware stores can be particularly useful for making small in‐house repairs. A set screw may cost a few cents, whereas sending a saw out for maintenance will cost appreciably more than that. If your institution has an instrument or machine shop, staff members can often help find cheaper solutions than through scientific vendors or may be able to repair supplies themselves. Be sure that any in‐house changes you make to purchased equipment do not void any warranties, purchased, or otherwise.

Craft stores are good places to find small quantities of adhesives, fabrics, clay, and materials to make and repair models.

#### Creating new items from scratch

If a completely unique product is warranted, then making it in‐house may be an affordable option, provided your institution has the necessary equipment and a little time. Glove boxes, trays, anatomical models, simulators, and even some dissection instruments can be custom created for a laboratory.^59^, ^60^, ^61^, ^62^

Items can either be created through subtractive or additive manufacturing. Subtractive manufacturing can be used for metal, ceramic, and wood products and is commonly called milling.^63^ Milling is very useful, but the equipment required typically limits it to machine shops. However, if a metal item is needed for durability or for sterilization, then finding a shop is often still the best way to get a custom item.

Additive manufacturing involves creating an item from the ground up, or adding components to an existing item. One of the more common means of additive manufacturing is 3D printing. This is an additive manufacturing process where plastic filament is melted and added in layers to form a shape. There are several different types of plastic filament, each with different durability, cost, and ease of use considerations.^64^

3D printing has some advantages over milling in terms of cost and skill required. A benefit of additive manufacturing is cost. Plastic filament is quite affordable at a few cents per gram and plastic items tend to be lightweight. In addition, typically the only out‐of‐pocket cost is for the material that goes into the final product, whereas milling usually requires payment for the full block of material (much of which will be wasted).

Many institutions and libraries already have 3D printers that can be used for a small fee, eliminating the need to purchase expensive equipment. If your laboratory's needs are great enough, purchase of a 3D printer may be justifiable, and there are many affordable introductory‐level models available. The College of Engineering at your institution may have resources to help model structures, or directly modify or repair existing instruments as well.

One challenge to 3D printing is that someone needs to learn to use 3D modeling software in order to design the part that the laboratory needs.^65^ There are free programs that provide reasonable utility for beginners, but drafting is a skill—like drawing—that needs to be practiced to create professional‐looking results. If you are modifying or repairing an item, a 3D scan of the item can be created for importing into the draft software. Dedicated 3D scanners can accomplish this, but most smartphones support apps that will use the phone camera to make the scan. Then, the scan can be imported into drafting software to adjust size or edit elements of the item to fit. The drafting software can then export the model into a format for printing. The export consists of directions for printing each layer or “slice” of the model and is commonly referred to as “slicing.” The actual printing of the item can take anywhere from a few minutes to many hours, depending on the size, complexity, and resolution of the item.

The major benefit of 3D printing is the ability to create a custom item faster and cheaper than to have a part shipped. The major downside is that the person doing the 3D printing is both the designer and manufacturer, and so research and development time and costs are on them.^66^

### Sustainability

Sustainability is an important consideration in the operation of all laboratory environments, human anatomy laboratories not exempted, for environmental stewardship as well as for budgetary reasons. The decision if any given resource should be single‐use versus reusable, or purchased new versus repurposed, has implications for cost, safety, logistics, and regulatory compliance.

#### Physical laboratory resources: Single vs. reuseable, permanent vs. short‐term

Over the last century, biological laboratories, including human anatomy dissection laboratories, have increasingly shifted toward single‐use, disposable items to minimize contamination risk and to streamline workflows. Because these disposable products are typically less expensive than more durable alternatives, procurement generally prefers approving single‐use buys. However, their environmental impact is likely greater, strengthening the argument for transitioning to durable options when cost over time is considered.

For some items, single‐use remains entirely defensible and necessary. Gloves, for example, cannot be reused safely. However, some materials from dissection laboratories must be discarded through biohazardous waste streams—typically at the laboratory's expense. This creates a hidden expense that increases the laboratory's overall cost. If waste volume increases substantially or disposal fees rise, the value‐to‐cost balance may shift. Another traditionally single‐use item is the laboratory gown, which could be replaced with fabric alternatives. Doing so, however, introduces new costs: purchasing and maintaining a washing machine, buying detergent, and allocating labor for laundry. Despite these additional requirements, the overall environmental resource investment remains lower than that for disposable gowns. Ultimately, these decisions are evolving and must be evaluated on a case‐by‐case basis.

Many items in any laboratory, such as dissection instruments, tables, and bins, are inherently reusable and can last indefinitely with proper care. These items require routine maintenance to remain safe and functional, and investing in infrastructure to support this care can yield savings in time, energy, and costs. For example, dissection instruments must be cleaned regularly. Handwashing them is often inefficient and labor‐intensive. While purchasing and installing a dishwasher involves upfront costs and logistical challenges, the ability to sanitize large batches of instruments at once can save time, reduce labor, and lower long‐term environmental impact.

Wherever practical and possible, for both environmental and financial sustainability, consider the following strategies:

- **Using existing institutional resources:** If your institution already has equipment, such as a dishwasher in a nearby laboratory, explore whether you can share it occasionally.
- **Repurpose items:** Many items can serve multiple roles. For example, head bolsters can be reused each semester to support donors at the neck, shoulders, and limbs and to hold instruments where they remain visible.
- **Choose durable over disposable:** Durable goods typically have a lower environmental impact and, over time, cost less than disposables—provided they are maintained. When disposables are unavoidable, select options that minimize waste. For instance, stainless steel scalpel handles with replaceable blades generate less waste and are usually more cost‐effective than single‐use scalpels with plastic handles.
- **Choose permanent over temporary:** Invest in items that are adaptable across multiple courses, including those your institution may offer in the future. This reduces the need for frequent purchases and prevents items from becoming obsolete.

### Care considerations

#### Differently embalmed human anatomy donor care

All human remains are susceptible to rapid decay, and so in the overwhelming majority of circumstances, it is ideal to preserve tissues for anatomical dissection. Proper preservation of tissues involves thorough fixation and disinfection.^67^ Fixation refers to the denaturation and cross‐linkage of proteins in the body, which stabilizes tissues and denatures enzymes that may aid in decomposition of tissues.^68^

The most common fixative in embalming, in the anatomy laboratory as well as in the funeral home, is formaldehyde (and related chemicals: paraformaldehyde and formalin). This potent, volatile chemical is popular for its reliable and long‐lasting fixative qualities, its antifungal and antimicrobial properties, and its ease of use.^67^ Rarely is formaldehyde used by itself in embalming; typically, it is mixed with phenol, alcohol, wetting agents, and/or EDTA. High‐volume and high‐pressure embalming with a formaldehyde‐based solution reliably ensures an embalming preparation that can last years at room temperature with proper care.

Unfortunately, formaldehyde has major disadvantages. Tissues embalmed using formaldehyde are “hard‐fixed” and will not regain suppleness with application of any other solutions. Joints are fixed in place permanently, reducing the donor's utility to physical therapy laboratories. Tissues' color turns gray‐beige, texture firmness and pliability are appreciably different from those in living tissues, and discolorations present in the donor will remain in place after embalming.^55^

Further, formaldehyde exposure to other living systems will come to their detriment. It is known for being a carcinogen; being an eye, nose, skin, and throat irritant; and for having an uncomfortably strong odor. Prolonged exposure to formaldehyde is a known occupational hazard, and staff and faculty exposure has been a concern in the anatomy laboratory for decades.^35^, ^67^ In addition, tissues fixed with formaldehyde may affect soil and water quality local to a burial site.^69^ Since human anatomy donors are so much more heavily saturated with formaldehyde‐based solutions than a typical decedent prepared for viewing or a wake, many cemeteries may decline to receive human anatomy donors for whole‐body burial. Instead, human anatomy donors are commonly cremated. Dissectors and embalmers in anatomy laboratories also often find working with formaldehyde unpleasant, as even in laboratories with high air turnover, the chemical smell may linger in hair and clothes and may be difficult to wash off if not addressed quickly.

### Non‐human whole body replica care

**Virtual Dissection Tables:** Virtual dissection tables are large, dissection table sized computers with a flatscreen monitor facing up, allowing students to look down upon the table and interact with the screen in similar ways as with a human anatomy donor. These tables are installed with proprietary software that may or may not be editable to include new structures (from MRI or human anatomy scans), show anatomical variation, radiology and cross‐section images, isolated structures, and body systems. They often come with built‐in quizzes and have the capacity for instructors to create their own. The virtual dissection table may be more or less intuitive to different users and may depend on stable, functioning wi‐fi.

**Synthetic Anatomical Models:** These are high‐fidelity models made out of silicone. Structures included are made with colored silicone and hard plastic. Plastic is used for rigid structures such as the vertebrae, teeth, and ribs. Soft silicone is used for other organs such as the skin, spleen, liver, lungs with breathable interior, muscles, nerves, arteries, veins, heart, kidneys, pancreas, uterus, ovaries, vagina, and anus. Models are submerged in manufacturer‐determined solutions in a stainless steel immersion table and are covered in antimicrobial towels for the face and torso. When the synthetic anatomical model is not being used, it is kept fully submerged in the table's built‐in chamber to ensure longevity. The solution itself needs to be changed periodically, requiring a nearby water supply and additional room for temporary anatomical model storage. Manufacturer instructions should be followed closely to avoid damage to the unit. The synthetic anatomical model table makes a low humming noise from a constantly working pump, which is minimal, but some students may find it distracting.

Both of these alternatives to human anatomy donors are sustainable options for laboratories that cannot support housing or funding of human anatomy donors.^70^ They may serve as useful supplements to traditional human anatomy laboratories, as well, giving instructors opportunities for demonstrating cross‐sectional anatomy next to or very close to a human anatomy donor with related dissection, and allowing students to interact with a greatly simplified version of the anatomy they are about to dissect.

They do require careful consideration before purchase, however. Neither are inexpensive, and both may require routine mandatory maintenance or subscription and/or licensing fees. They both are at least as large as a standard dissection table, with the synthetic anatomical model usually being closer in size to a dip‐tank table. Similar to dissection tables, infrastructural considerations need to be made when placing and running each item (e.g., sink, water supply, power outlets). Both must be kept in a “clean” area of the laboratory—both have potential to be quickly damaged if contaminated by oils or fluids from human anatomy donors (and this type of damage may not be covered under warrantee or insurance policies).

And finally, neither offer the same opportunities for tactile interaction, rehearsal of surgical skills, or appreciation for anatomical variation and interspatial relationship of structures in three dimensions to a true‐to‐life level of detail. Importantly, neither address “hidden curriculum” elements—educational themes essential to basic professional education but otherwise not directly addressed, such as coping with and addressing death or preserving patient dignity.^6^

## CONCLUSION

The task of creating or maintaining a human anatomy dissection laboratory presents unique challenges and opportunities that require care and attention to detail not found in other laboratory spaces. Variables such as different building spaces, budgets, institutional or state requirements, cohort sizes, space use demands, embalming styles, and existing infrastructure all dictate what can be done with any specific space. Each variable is under different levels of individual control. Ultimately, each decision should be made regarding what is best for any given laboratory's needs, regardless of whether it is mentioned in this Guide. The ADS can provide a supplementary Handbook, which is intended to be a living document. It may be updated to include your creative solutions and shared with you to support the management of your laboratory space.

## AUTHOR CONTRIBUTIONS

**Rhiannon E. Robinson:** Writing – original draft; conceptualization; writing – review and editing. **Burhan Gharaibeh:** Writing – original draft; writing – review and editing. **Molly Ostwald:** Writing – original draft; writing – review and editing. **Melissa Zolnierz:** Writing – review and editing; writing – original draft. **Jonathan J. Wisco:** Conceptualization; writing – original draft; writing – review and editing. **Bobbie J. Leeper:** Conceptualization; writing – original draft. **Jeremy J. Grachan:** Conceptualization; writing – original draft; writing – review and editing. **Mary Schilling:** Writing – original draft; writing – review and editing. **Abbey Breckling:** Writing – original draft; conceptualization; writing – review and editing. **Kelsey Stevens:** Conceptualization; writing – original draft. **Danielle N. Edwards:** Conceptualization; writing – original draft; writing – review and editing. **J. P. Swigart:** Writing – original draft; writing – review and editing. **Bridgett Severt:** Writing – original draft; writing – review and editing. **Nicole Geske:** Writing – original draft; writing – review and editing.

## Data Availability

Data sharing not applicable to this article as no datasets were generated or analysed during the current study.

## References

[1] Ghosh SK . Human cadaveric dissection: a historical account from ancient Greece to the modern era. Anat Cell Biol. 2015;48(3):153–169. Available from: https://synapse.koreamed.org/articles/1071811 10.5115/acb.2015.48.3.153 PMC4582158 26417475

[2] Winkelmann A . Anatomical dissection as a teaching method in medical school: a review of the evidence. Med Educ. 2007;41(1):15–22. 10.1111/j.1365-2929.2006.02625.x 17209888

[3] Fornai F , Alfonso Arráez‐Aybar L . Evolving anatomy education: bridging dissection, traditional methods, and technological innovation for clinical excellence. Anatomia. 2025;4(2):9. 10.3390/anatomia4020009

[4] MacPherson E , Lisk K . The value of in‐person undergraduate dissection in anatomical education in the time of Covid‐19. Anat Sci Educ. 2022;15(4):797–802. 10.1002/ase.2186 PMC9348114 35523727

[5] Beckman E , Childers CE , Hartsock J . A missed opportunity: humanities in anatomy lab. J Med Humanit. 2025;46(4):719–727. 10.1007/S10912-025-09937-6 40019727

[6] Kumar Ghosh S , Kumar A . Building professionalism in human dissection room as a component of hidden curriculum delivery: a systematic review of good practices. Anat Sci Educ. 2019;12(2):210–221. 10.1002/ASE.1836 30376608

[7] Goldman E . Building a low‐cost gross anatomy laboratory: a big step for a small university. Anat Sci Educ. 2010;3(4):195–201. 10.1002/ase.160 20568286

[8] Trelease RB . Anatomy meets architecture: designing new laboratories for new anatomists. Anat Rec B New Anat. 2006;289(6):241–251. 10.1002/ar.b.20118 17109425

[9] Wessels Q , Vorster W , Jacobson C . Anatomy education in Namibia: balancing facility design and curriculum development. Anat Sci Educ. 2012;5(1):41–47. 10.1002/ase.1250 22213639

[10] Zdilla MJ . Creating a human gross anatomy laboratory: the experience at a primarily undergraduate institution. Anat Sci Educ. 2020;13(5):636–647. 10.1002/ase.1946 31990441

[11] Gabriel AG , Chia SL . High price of perfection that is anatomy: why studying and teaching the human body is a financial muscle. J Innov Med Res. 2025;4(2):84–88.

[12] Handady G , Dsouza A , Nayak V , Abraham J . Formaldehyde levels and the indoor air quality of an anatomy dissection hall with different ventilation setups. Environ Health Insights. 2024;18:11786302241301590. 10.1177/11786302241301590 PMC11603455 39610457

[13] Zdilla MJ . Local exhaust ventilation systems for the gross anatomy laboratory. Morphologie. 2021;105(350):237–246. 10.1016/j.morpho.2020.11.002 PMC8169711 33279395

[14] Greene JRT . Design and development of a new facility for teaching and research in clinical anatomy. Anat Sci Educ. 2009;2(1):34–40. 10.1002/ase.70 19217068

[15] Mayfield CH , Ohara PT , O'Sullivan PS . Perceptions of a mobile technology on learning strategies in the anatomy laboratory. Anat Sci Educ. 2013;6(2):81–89. 10.1002/ase.1307 22927203

[16] American Association for Anatomy . Gross anatomy lab design. n.d. Available from: https://www.anatomy.org/ANATOMY/ANATOMY/Resources/Gross‐Anatomy‐Laboratory‐Design.aspx

[17] Human Anatomy & Physiology Society . Human body lab design process. n.d.‐a. Available from: https://membership.hapsweb.org/general/custom.asp?page=HumanBodyLabDesign

[18] Darras KE , Spouge R , Hatala R , Nicolaou S , Hu J , Worthington A , et al. Integrated virtual and cadaveric dissection laboratories enhance first year medical students' anatomy experience: a pilot study. BMC Med Educ. 2019;19(1):366. 10.1186/s12909-019-1806-5 PMC6781397 31590672

[19] Neyem A , Cadile M , Burgos‐Martínez SA , Farfán Cabello E , Inzunza O , Alvarado MS , et al. Enhancing medical anatomy education with the integration of virtual reality into traditional lab settings. Clin Anat. 2025;38(3):249–268. 10.1002/ca.24213 39245875

[20] Reeves RE , Aschenbrenner JE , Wordinger RJ , Roque RS , Sheedlo HJ . Improved dissection efficiency in the human gross anatomy laboratory by the integration of computers and modern technology. Clin Anat. 2004;17(4):337–344. 10.1002/ca.10245 15108341

[21] Department of Health & Human Services . Biosafety Levels & Lab Safety Guidelines. n.d. Available from: https://aspr.hhs.gov/S3/Pages/Biosafety‐Level‐Requirements.aspx. Accessed 12 Dec 2025.

[22] Leeper BJ , Grachan JJ , Robinson R , Doll J , Stevens K . Honoring human body donors: five core themes to consider regarding ethical treatment and memorialization. Anat Sci Educ. 2024;17(3):483–498. 10.1002/ase.2378 38197550

[23] Rizzolo LJ . Human dissection: an approach to interweaving the traditional and humanistic goals of medical education. Anat Rec. 2002;269(6):242–248. 10.1002/ar.10188 12467081

[24] Sv S , Maria Francis Y , Karunakaran B , Raghunath G , Kumaresan M , Begum Z , et al. Donor oath: respect to the mortal teacher to learn ethics and humanitarian values of anatomy. Cureus. 2022;14(3):e22941. 10.7759/cureus.22941 PMC8986947 35411283

[25] Weeks SE , Harris EE , Kinzey WG . Human gross anatomy: a crucial time to encourage respect and compassion in students. Clin Anat. 1995;8(1):69–79. 10.1002/ca.980080113 7697517

[26] Onigbinde OA , Ajagbe AO . Handling asthmatic students in the dissection hall. Ulutas Med J. 2021;7(3):145.

[27] Pabst R . Exposure to formaldehyde in anatomy: an occupational health hazard? Anat Rec. 1987;219(2):109–112. 10.1002/ar.1092190202 3688466

[28] Haffner MJ , Oakes P , Demerdash A , Yammine KC , Watanabe K , Loukas M , et al. Formaldehyde exposure and its effects during pregnancy: recommendations for laboratory attendance based on available data. Clin Anat. 2015;28:972–979. 10.1002/ca.22623 26375478

[29] Collins JJ , Ness R , Tyl RW , Krivanek N , Esmen NA , Hall TA . A review of adverse pregnancy outcomes and formaldehyde exposure in human and animal studies. Regul Toxicol Pharmacol. 2001;34(1):17–34. 10.1006/rtph.2001.1486 11502153

[30] Hammer N , Stöger K , Antipova V . Formaldehyde use in the anatomy laboratory and the pregnant or breastfeeding student dissector navigating the challenges of toxicological uncertainty and institutional policy. Ann Anat. 2026;266:152813. 10.1016/j.aanat.2026.152813 41759790

[31] Wozniak K , Fagan R , Krause M . Dissecting the impact of anatomy lab policies on pregnant medical students. Clin Anat. 2025;38(6):706–711. 10.1002/ca.70005 40605446

[32] Washmuth NB , Cahoon T , Tuggle K , Hunsinger RN . Virtual dissection: alternative to cadaveric dissection for a pregnant nurse anesthesia student. Health Prof Educ. 2020;6(2):247–255.

[33] Hayman J , Oxenham M . Human body decomposition. London: Elsevier; 2016.

[34] Anapath . Material Safety Data Sheet Section 1. Chemical product and company identification. n.d. Available from: https://www.aphis.usda.gov/sites/default/files/MSDS_Formalin.pdf. Accessed 11 Dec 2025.

[35] OSHA . 1910.1048 App A—Substance Technical Guidelines for Formalin | Occupational Safety and Health Administration. 2013 Feb. Available from: https://www.osha.gov/laws‐regs/regulations/standardnumber/1910/1910.1048AppA

[36] Cameo Chemicals . Formaldehyde, solution, flammable. n.d. Available from: https://cameochemicals.noaa.gov/chemical/769. Accessed 1 Apr 2026.

[37] Occupational Safety and Health Administration . (2011) Laboratory Safety Guidance. (OSHA 3404‐11R). U.S. Department of Labor. osha.gov

[38] FDA/NIOSH . Sharps Disposal Containers in Health Care Facilities | FDA. 2021 Apr. Available from: https://www.fda.gov/medical‐devices/safely‐using‐sharps‐needles‐and‐syringes‐home‐work‐and‐travel/sharps‐disposal‐containers‐health‐care‐facilities

[39] Hasan F , Shah AA , Javed S , Hameed A . Enzymes used in detergents: lipases. Afr J Biotechnol. 2010;9(31):4836–4844. 10.5897/AJBx09.026

[40] Schwope AD , Costas PP , Mond CR , Nolen RL , Conoley M , Garcia DB , et al. Gloves for protection from aqueous formaldehyde: permeation resistance and human factors analysis. Appl Ind Hyg. 1988;3(6):167–176. 10.1080/08828032.1988.10390258

[41] Mahler V , Fischer S , Fuchs T , Ghannadan M , Valent P , Fartasch M , et al. Prevention of latex allergy by selection of low‐allergen gloves. Clin Exp Allergy. 2000;30(4):509–520. 10.1046/J.1365-2222.2000.00721.X 10718848

[42] ANSI Standards . ANSI/ISEA Z87.1‐2020: Current Standard for Safety Glasses—The ANSI Blog. n.d.‐b. Available from: https://blog.ansi.org/ansi/ansi‐isea‐z87‐1‐2020‐safety‐glasses‐eye‐protection/. Accessed 11 Dec 2025.

[43] ANSI Standards . ANSI Z358.1‐2014: Learn About the ANSI Eyewash Standard—ANSI Blog. n.d.‐a. Available from: https://blog.ansi.org/ansi/ansi‐z358‐1‐emergency‐eyewash‐station‐shower/. Accessed 11 Dec 2025.

[44] Lee YH , Lee Y‐M , Kwon S , Park S‐H . Reactions of first‐year medical students to cadaver dissection and their perception on learning methods in anatomy. Korean J Med Educ. 2011;23(4):275–283. 10.3946/KJME.2011.23.4.275 PMC8813518 25812687

[45] Aydin MA , Salukhe TV , Wilke I , Willems S . Management and therapy of vasovagal syncope: a review. World J Cardiol. 2010;2(10):308–315. 10.4330/WJC.V2.I10.308 PMC2998831 21160608

[46] Human Anatomy & Physiology Society . Student accommodations handbook. n.d.‐b. Available from: https://www.hapsweb.org/teaching‐resource‐hub/student‐accommodations‐handbook/

[47] Norval GW . Chronic lung impact on laboratory worker exposed to chloramines and cyanogen chloride. ACS Chem Health Saf. 2020;27(2):129–132. 10.1021/ACS.CHAS.9B00020

[48] Prince R , Monsen JR , Webber Z , Olsen AL , Nelson T , Reeves CJ , et al. Staying hydrated—a comparative analysis of humectants in human cadaveric tissue. HAPS Educ. 2025;29(4):5–13. 10.21692/haps.2025.023

[49] Langroudi MF , Farzanegan A . Decrease of microbial contamination by application of routinely glycerol and phenol solution on human cadavers. J Curr Biomed Rep. 2021;2:74–78.

[50] Chen HJ , Lee PY , Chen CY , Huang SL , Huang BW , Dai FJ , et al. Moisture retention of glycerin solutions with various concentrations: a comparative study. Sci Rep. 2022;12(1):10232. 10.1038/S41598-022-13452-2 PMC9205919 35715536

[51] Poddębniak P , Kalinowska‐Lis U . A survey of preservatives used in cosmetic products. Appl Sci. 2024;14(4):1581. 10.3390/APP14041581

[52] Blaney SP , Johnson B . Technique for reconstituting fixed cadaveric tissue. Anat Rec. 1989;224(4):550–551. 10.1002/ar.1092240413 2782634

[53] Krishnamurthy S , Powers SK . The use of fabric softener in neurosurgical prosections. Neurosurgery. 1995;36(2):420–424. 10.1227/00006123-199502000-00029 7731528

[54] Balta JY , Cronin M , Cryan JF , O'Mahony SM . Human preservation techniques in anatomy: a 21st century medical education perspective. Clin Anat. 2015;28(6):725–734. 10.1002/CA.22585 26118424

[55] Hunter A , Eisma R , Lamb C . Thiel embalming fluid—a new way to revive formalin‐fixed cadaveric specimens. Clin Anat. 2014;27(6):853–855. 10.1002/CA.22392 24639210

[56] Thompson B , Green E , Scotcher K , Keenan ID . A novel cadaveric embalming technique for enhancing visualisation of human anatomy. Adv Exp Med Biol. 2022;1356:299–317. 10.1007/978-3-030-87779-8_13 35146627

[57] Turan E , Gules O , Kilimci FS , Kara ME , Dilek OG , Sabanci SS , et al. The mixture of liquid foam soap, ethanol and citric acid as a new fixative–preservative solution in veterinary anatomy. Ann Anat. 2017;209:11–17. 10.1016/J.AANAT.2016.09.002 27743852

[58] Hammer N , Löffler S , Feja C , Sandrock M , Schmidt W , Bechmann I , et al. Ethanol‐glycerin fixation with thymol conservation: a potential alternative to formaldehyde and phenol embalming. Anat Sci Educ. 2012;5(4):225–233. 10.1002/ASE.1270 22434588

[59] Ford J , Goldstein T , Trahan S , Neuwirth A , Tatoris K , Decker S . A 3D‐printed nasopharyngeal swab for COVID‐19 diagnostic testing. 3D Print Med. 2020;6(1):21. 10.1186/S41205-020-00076-3 PMC7429130 32803531

[60] George M , Aroom KR , Hawes HG , Gill BS , Love J . 3D printed surgical instruments: the design and fabrication process. World J Surg. 2017;41(1):314–319. 10.1007/S00268-016-3814-5 PMC6287965 27822724

[61] Suzuki M , Watanabe R , Nakazono A , Nakamaru Y , Suzuki T , Kimura S , et al. Can high‐fidelity 3D models be a good alternative for cadaveric materials in skill assessment for endoscopic sinus surgery? A comparison study in assessment for surgical performance in 3D models and cadavers. Front Med. 2024;11:1301511. 10.3389/FMED.2024.1301511 PMC11524814 39484199

[62] Wong JY , Pfahnl AC . 3D printing of surgical instruments for long‐duration space missions. Aviat Space Environ Med. 2014;85(7):758–763. 10.3357/ASEM.3898.2014 25022166

[63] Alghazzawi TF . Advancements in CAD/CAM technology: options for practical implementation. J Prosthodont Res. 2016;60(2):72–84. 10.1016/J.JPOR.2016.01.003 26935333

[64] Mcmenamin PG , Quayle MR , Mchenry CR , Adams JW . The production of anatomical teaching resources using three‐dimensional (3D) printing technology. Anat Sci Educ. 2014;7(6):479–486. 10.1002/ASE.1475 24976019

[65] Raechal L , Bajwa M , Fayyaz J , Biglino G , Kardong‐Edgren S . A scoping review of literature about 3D printing: knowledge, skills and attitude for simulation educators in healthcare. 3D Print Med. 2025;11(1):46. 10.1186/S41205-025-00292-9 PMC12376359 40853422

[66] Walker V . Implementing a 3D printing service in a biomedical library. J Med Libr Assoc. 2017;105(1):55–60. 10.5195/JMLA.2017.107 PMC5234447 28096747

[67] Gee‐Mascarello SL , Mayer RG . Embalming: history, theory, and practice. New York: McGraw‐Hill; 2022.

[68] Eltoum I , Fredenburgh J , Myers RB , Grizzle WE . Introduction to the theory and practice of fixation of tissues. J Histotechnol. 2001;24(3):173–190. 10.1179/HIS.2001.24.3.173

[69] Lima BBM e R , Marini AF , Manhães AJP , Sales D d S , Guimarães HA , Sales CMR , et al. Cemeteries and environmental contamination: strategies for control, monitoring and remediation. Rev Gestao Soc Ambient. 2025;19(10):e013549. 10.24857/RGSA.V19N10-018

[70] Verma S , Sharma U . Alternatives to cadaveric dissection in medical education: an overview. Int J Adv Med Health Res. 2025;12(1):12–22. 10.4103/IJAMR.IJAMR_316_24

